# Understanding the Economic Value and Impacts on Informal Carers of People Living with Mental Health Conditions

**DOI:** 10.3390/ijerph19052858

**Published:** 2022-03-01

**Authors:** David McDaid, A-La Park

**Affiliations:** Care Policy and Evaluation Centre, Department of Health Policy, London School of Economics and Political Science, London WC2A 2AE, UK; a.park@lse.ac.uk

**Keywords:** informal care, mental disorders, cost of illness, caregivers, loneliness

## Abstract

Informal carers play a vital role in supporting people living with mental health conditions, but comparatively little is known about the economic value of caring. This study undertook an online survey of adult informal carers supporting adults with mental health conditions to better understand the impacts of caring on carer quality of life, levels of loneliness, finances and employment, as well as estimate the economic value of time spent caring. In total, 712 carers participated in the multi-national survey between August 2019 and April 2020. A total of 17% were male, with a mean age of 53, and 68% supported a child living with a mental health condition. A total of 56% of care recipients were male, with a mean age of 37. Adverse impacts on quality of life, loneliness and personal finances were greatest in carers living with care recipients. Overall mean weekly hours of care were 43.42, rising to 65.41 for carers living with care recipients. Mean weekly costs of care per carer ranged from €660 to €2223 depending on living arrangements. Annual costs ranged between €34,960 and €125,412, depending on living arrangements and valuation method. Informal care costs are substantial, and policy makers should consider investing more in carer support, especially for carers living with care recipients.

## 1. Introduction

Family and other informal caregiving is a fundamental contribution made to the welfare of all societies. Throughout life, we all rely on different levels of care and support from family and friends. Some of this caring is seen as a fundamental duty, this being most evident for parents providing care for their children until they reach adulthood. In this case, many high-income countries, recognising the societal value of this care, provide financial help and other supports to help parents manage their caring responsibilities alongside some of their other activities such as going to work.

For other groups of carers, including informal carers supporting people with mental health conditions, the level of recognition of the value of care and support for carers from policy makers appears much more limited. For example, more than a decade ago, a parliamentary report looking at the needs of carers supporting people with mental health conditions in Canada stated that “caregivers feel excluded, ignored by the mental health, mental illness and addiction system in Canada. Ironically, it is these same family members who often provide most of the care and support to people living with mental illness” [1]. In many European countries, carers also appear to receive little support for their own health and welfare needs [2].

From a public health perspective, informal, unpaid carers, usually family members, are an essential part of mental health systems, supporting people immediately during mental health crises, as well as over the longer term to promote recovery and social inclusion. The number of carers is noteworthy; in Australia, in 2018, for example, approximately 1.4% of the population were providing informal care to adults with mental health conditions [3]. If governments are to invest more in supporting informal carers, then it is critical to better identify and value, from an economic perspective, the rewards and challenges associated with providing informal care to people living with mental health conditions.

There is a rich literature base on the economics of informal care. Much of this literature focuses on quantifying and valuing time spent caring [4]. Time spent on informal caregiving is time that is no longer available for other activities. These ‘opportunity costs’, including time lost from employment, volunteering and leisure, make up the bulk of the immediate economic impacts of informal caring; if carers were unwilling or unable to provide their time for informal care, then some, if not all of these costs, would fall on health and social care services. For example, analysis in Sweden estimated that the annual cost of informal care for all health conditions is equivalent to 3% of the Swedish Gross Domestic Product (GDP); it was also estimated that replacing this informal care with professional care would be even more expensive at 4% of GDP [5]. A Europe-wide analysis also suggested that the annual economic value of all caregiving is equivalent to 3.63% of the European GDP [6].

In the case of very well studied conditions such as dementia, where informal caring can be a 24 hour, 7 day, activity, costs for individual carers can be very substantial indeed [7]. As caring becomes a more intensive time-consuming activity, carers, particularly women and those living with care recipients, may end up withdrawing entirely from the labour force or education, as well as retiring early [8,9]. In addition, there are well-documented impacts on carer mental and physical health and quality of life [10,11]. They are also at greatest risk of being lonely than non-carers [12]; loneliness itself is a risk factor for further deterioration in carer physical [13] and mental health [14]. Quality of life is also important to consider as it is a key metric used by policy makers when considering the cost effectiveness of health care interventions in many European countries [15] and in Canada [16].

While reasonably well documented in respect to dementia, the economic value and impacts of informal care are still not well understood when looking at other mental health problems including anxiety and depression, bipolar disorder and psychoses [17]. Studies tend to be small in size and scope, focusing on carers supporting individuals with a single condition only. For example, one study in Spain highlighted that informal care accounted for 47% of all costs of care for schizophrenia [18], with a similar finding in a small study in Germany [19]. Another study in Italy modelled the annual costs of schizophrenia; however, it only included mean costs of days out of normal roles [20]. The most detailed analysis from Australia looked at time spent caring by family carers for a range of mental health conditions. It estimated that primary carers provide an average of approximately 36 h of care per week, with 38% caring for 40 or more hours per week [3,21,22]. Surprisingly, even with increased interest in the experiences of carers during the COVID-19 pandemic, and much evidence on the adverse impacts on carer physical and mental health [23,24], there does not appear to have been a focus in these studies on the economic value of caring for all carer groups, let alone for carers supporting people with mental health conditions.

Given the limited information available on the economic value of caring, we designed and conducted a survey in collaboration with the non-governmental organisation, the European Federation of Associations of Mentally Ill People (EUFAMI), to better estimate the economic value of time spent caring, as well as to understand the economic impacts of caring on carer quality of life, levels of loneliness, health, finances and employment.

## 2. Materials and Methods

### 2.1. Survey Development

An online survey was developed to collect a broad range of information on the experience of being an informal carer (see Supplementary Materials for English version of survey). To be eligible to participate, respondents had to be 18 or older and caring for someone aged 18 or older with at least one severe mental health condition (other than dementia and learning difficulties). They did not have to be the main informal carer, nor live in the same household as the person they cared for. Paid carers were not eligible to participate.

The survey was targeted at eight high-income countries where EUFAMI members were willing to help raise awareness of the survey, as well as help with translation: Canada, Denmark, France, Ireland, Italy, Malta, Spain and the UK. This choice of countries meant that we were able to have representation from the main social welfare systems across Western Europe [25]: the Nordic model with universal coverage for social services and generous social welfare benefits (Denmark), the Anglo-Saxon model which typically targets services and welfare benefits at those most in need (Ireland, UK), the Continental model which has universal coverage but mostly through financial support rather than services (France) and the Mediterranean model (Spain, Italy) with less extensive services and welfare benefits.

A convenience sampling approach was adopted, with the survey relying on EUFAMI country partners using different approaches to raise awareness of the survey locally; this included social media posts on Twitter and Facebook as well as use of mailing lists. In addition, the EUFAMI also raised awareness of the survey through posts on its own website. Respondents from beyond these eight countries were also free to take part in the survey but no active efforts were made to recruit these additional carers.

The survey was administered using QUALTRICS, a secure online survey collection system. It was launched online in August 2019, in English, French, Spanish, Italian and Danish. Different country specific versions of the survey were available in English to cover the different contexts in the UK, Ireland, Canada and Malta. There were also two French versions for Canada and France. Respondents were free to skip questions and not answer any questions they did not wish to answer. This study received ethical approval through the Personal Social Services Research Unit’s ethics process in line with the LSE’s Research Ethics Policy and Procedure on February 8, 2019. All responses were anonymous and informed consent was required to participate in the survey.

### 2.2. Survey Content

The survey questionnaire included key metrics needed to accurately value informal care including a carer-specific quality of life instrument originally developed in the Netherlands, the CarerQoL-7D, and tested and validated in a number of European countries, to measure the impact of caring on carer quality of life [26,27]. The instrument has seven domains reflecting different aspects of caring, including fulfilment from caring, relational problems with the person being cared for, mental and physical health impacts, difficulty managing care tasks, financial impacts and access to support. Separately, happiness was measured with a single item question with scores ranging from 0 (least happy) to 100 (most happy) (see survey in Supplementary Materials to see full instrument). Country specific weightings (tariffs) are attached to the different levels of each domain in order to calculate a quality of life score, where 0 represents the worst possible carer quality of life and 100 indicates that caring has no detrimental impacts on carer quality of life.

Carers are also known to be at higher risk of loneliness compared to the general population [28,29], but there has been limited research looking at the experience of informal carers of people with mental health problems. We therefore looked at the potential association between experiences of caring and levels of loneliness, measured using the brief 3-item version of the University of California, Los Angeles (UCLA-3) loneliness scale [30]. Scores range from 3 (least lonely) to 9 (most lonely).

In order to estimate and value the amount of time spent providing care, survey respondents were also asked to indicate how many hours per week they spent providing care using a visual analogue scale running from 0 to 168 h per week. We also asked carers how much they would either be willing to pay (WTP) for someone else to provide an extra hour of caring tasks or how much money they would be willing to accept (WTA) to provide an extra hour of caring tasks themselves. This methodology reflects the value placed on care by carers with lived experience of the caring process, rather than simply estimating the replacement costs if informal care were to be replaced by formal care provided at a specific hourly wage [31]. Another aspect of the value of caring is the travel costs associated with caring, particularly where carers live separately from the person they are caring for. Carers were also asked to estimate both weekly travel time and travel expenses. We added travel expenses, plus travel time valued using the WTA hourly rate, to the overall value of caring. Additionally, adapted versions of questions related to the financial burden of caring were drawn from a previous multi-country carer survey [32].

The monetary value of caring time represents only a part of the true value of care. In addition, there are also adverse impacts on quality of life. These impacts are not easy to value economically; many carers may even feel that it is inappropriate to try and put a monetary value on them. That said, it is common to place a monetary value on adverse impacts on quality of life. Here, we have assumed that impacts on quality of life are constant and, for illustrative purposes, conservatively valued each year of perfect quality of life using the CarerQoL-7D at €30,000. This annual value is similar to that seen in health economic studies in the UK and Spain [33,34]. Values were adjusted for purchasing power parity dependent on country of respondent and then reported in 2020 Euros (€). All other costs reported in this paper are also reported in purchasing power-adjusted 2020 Euros.

### 2.3. Statistical Analyses

We used all available cases for analyses, including data from partially completed questionnaires. We front loaded the questionnaire with demographic information followed by priority questions that would allow us to explore the economic impacts of care: hours of care per week, UCLA-3 loneliness scores, carer quality of life, WTA and WTP for an extra hour of care, and impacts on carer work hours.

Appropriate statistical analyses were applied to different survey response data. Independent-samples t-tests with 1000 bias-corrected and accelerated bootstraps were used to determine whether there were significant differences in quality of life, happiness and loneliness scores, hours of care provided, as well as WTA or WTP values for someone else to provide an extra hour of care for carers who live with the person they care for compared to carers who live separately from the person they care for. Generalised linear models (GLM) with gamma distribution and identity link were also used to explore the association between individual carer characteristics and loneliness. In our GLM models, we considered carer age, gender, number of hours caring per week, happiness, living arrangements and carer quality of life as potential factors that could impact on loneliness scores. One-way analysis of variance was used to assess whether there were significant differences in caring hours for individuals caring for someone with a single mental health condition compared to those caring for someone with multiple conditions. This analysis was also used to compare differences in caring hours by carer gender, and the relationship between carer and care recipient. For the latter, post hoc pairwise comparisons using the Tukey test were used to identify which, if any, differences in mean hours were significant.

## 3. Results

### 3.1. Profile of Carers

In total, 712 individuals gave their consent and participated in this study between 1 August 2019 and 1 April 2020. A total of 272 carers (38%) were from Denmark, followed by carers from France 152 (21%), Spain 115 (16%), Ireland 57 (8%), Canada 51 (7%) and the UK 40 (6%), with 25 (4%) from 13 other countries. On average, respondents completed 67% of the questionnaire, with 50% fully completing the questionnaire. Data were not missing at random; and although response rates were lower for later questions in the survey, there were no significant differences in missing data across countries or between carers by living arrangements.

Table 1 provides a summary of key carer characteristics for all carers, by different living arrangements. Only 17% of carers were male, with a mean age of 53.4. A total of 68% of carers were supporting a child living with a mental health condition. A minority, 42%, of carers lived with the person they supported. A total of 54% of carers were not in employment, including 26% who considered themselves retired. A higher proportion of carers who lived with the person they supported were more likely to be supporting a spouse and less likely to be supporting siblings, friends and other relatives. Most carers in the survey (64%) were ‘experienced’ carers who had been supporting someone with a mental health condition for more than 3 years; in contrast, only 7% of survey respondents had been caring for one year or less. Overall, 331 respondents (47%) were the sole unpaid carer, with a further 174 (24%) sharing care with others but being the primary carer. A total of 67% of carers living with the person they were supporting were the sole carer compared to 36% of carers who lived separately.

### 3.2. Profile of Care Recipients

Overall, 56% of care recipients were male (Table 2), with a mean age of 37.35. A total of 21% were aged 18 to 24 compared with 8% aged 65 or older. The mean number of reported mental health conditions per care recipient was 1.54. A total of 45% of care recipients had a psychotic condition (schizophrenia, schizoaffective disorder or other psychosis) and 29% had a mood disorder (major depression or bipolar disorder). A total of 24% of care recipients had an anxiety disorder (general anxiety disorder, panic disorder, obsessive compulsive disorder, post-traumatic stress disorder, as well as social or other phobia).

### 3.3. Impacts on Carers

We looked at impacts on quality of life using the CarerQoL-7D. As Table 3 shows, these data were available for 518 (73%) of all respondents. Country specific tariffs were applied and overall the mean quality of life score, measured as 100 = highest value and 0 = lowest value, is 60.17 for these 518 carers. Independent-samples t-tests with 1000 bias-corrected and accelerated bootstraps revealed that quality of life scores for carers who live with the person they care for are significantly lower than for carers who live separately from the person they care for (55.93 (95% CI 53.04 to 58.77) versus 63.75 ((95% CI 61.41 to 66.00) *p* = 0.001). The mean happiness score for 512 (72%) of all responding carers was 49.01. Happiness scores were also significantly lower for carers who live with the person they care for (43.71 (95% CI 40.81 to 46.74) versus 53.42 ((95% CI 50.80 to 55.97) *p* = 0.001).

In total, 520 (73%) of all carers completed the UCLA-3 loneliness measure. The mean loneliness score was 5.88. (Table 3). Independent-samples t-tests with 1000 bias-corrected and accelerated bootstraps revealed that carers who live with the person they care for have significantly higher levels of loneliness than carers who do not live with the person they support, mean scores of 6.56 (95% CI 6.30 to 6.81) versus 5.30 (95% CI 5.07 to 5.53, *p* = 0.001).

We also undertook a generalised linear regression analysis that looked further at potential factors that influence loneliness levels in 504 (71%) of all carers for whom data were available (Table 4). We found that there was an association between age of carer and levels of loneliness. For every one-year increase in age, loneliness scores decreased by 0.011 (*p* = 0.041). Perhaps one reason for this might be that older carers may be more experienced and have better developed coping strategies, and they may also have fewer other caring responsibilities than younger carers. When carers lived with the people they were caring for, this was associated with carers feeling more lonely (*p* = 0.009). Higher levels of happiness or quality of life were associated with lower levels of loneliness (*p* = 0.000). Increased caring time was also associated with higher levels of loneliness (*p* = 0.000).

Our survey also indicates that substantial numbers of carers report the adverse impacts of caring on their ability to participate in work. A total of 491 (69%) of all respondents provided information on changes in their hours of work because of their caring responsibilities, with 213 (43%) of these 491 carers reducing their hours in work. This rose to 48% for carers living with the person they cared for. On average, for the 213 carers who had reduced their working time, this was by 19.49 h a week, equivalent to more than half of a typical working week. Carers living with the person they cared for gave up an average of 21.05 h of work per week compared to 18.00 h per week for carers who lived separately. This difference was almost significant (*p* = 0.071).

A total of 235 (33%) of all carers responded to our survey question on carer finances. In total, 75 (32%) of these 235 respondents were at least moderately concerned about their finances due to caring, while a further 57 (24%) had great concerns about their finances. A total of 231 (32%) of all responding carers provided responses to questions on retirement income. Of these, 76 (33%) were concerned about their level of income in retirement due to reduced working opportunities. A total of 84 (36%) of these 231 carers stated that they had or would have to postpone their retirement because of their caring responsibilities. This rose to 43% for carers living with the care recipient.

### 3.4. Quantifying Caring Time

One key way in which the value of caring is measured is to estimate and value the amount of time spent providing care. In our survey, carers were asked to indicate how many hours per week they spent providing care using a visual analogue scale running from 0 to 168 h per week. Responses from 563 (79%) of all carers were recorded, providing an average of 43.42 h (SD 45.3) every week. Carers living with the person they support reported a significantly higher number of mean hours of care per week: 65.41 (SD 51.50) versus 25.71 (SD 29.80) hours per week. With 1000 bias-corrected and accelerated bootstraps, this was a significant mean difference of 39.71 h per week (95% CI 32.22—47.51) (*p* = 0.001).

One-way analysis of variance revealed no significant differences in caring hours either for individuals caring for someone with a single mental health condition, or between carers supporting people with different numbers of mental health conditions. There was also no significant difference in hours of care provided by 112 male carers (46.25) versus 445 female carers (42.96) (*p* = 0.50), but one-way analysis of variance indicated some significant differences in hours of care provided depending on the relationship between the carer and care recipient. Running post hoc pairwise comparisons using the Tukey test to identify which differences in mean hours were significant, we found that 67 carers supporting a partner/spouse provided significantly more mean weekly hours of care (61.52) than carers supporting a son or daughter (40.64) (mean difference 20.88, *p* = 0.009). This level of weekly hours of care is similar to that for carers that live with the person they care for, as is the case for most spousal carers. There were no other significant differences between different carer relationships although the higher number of hours for spousal carers was almost significantly greater than caring hours for parents or brother or sisters.

### 3.5. Valuing Caring Time

In this survey, we asked carers how much they would either be willing to pay for someone else to provide an extra hour of caring tasks or how much money they would be willing to accept provide an extra hour of caring tasks themselves. Table 5 reports mean willingness to pay (WTP) and willingness to accept (WTA) values per hour of informal care provision. A total of 522 (73%) of all respondents provided this information. Carers would on average have to receive €28.69 to provide one extra hour of care themselves; they would be willing to pay €23.62 for someone else to provide an extra hour of care. Mean WTA and WTP values for carers living with the care recipient were higher than for carers who lived separately from the care recipient, €30.44 and €25.07 versus €27.46 and €22.27, respectively. After 1000 bias-corrected and accelerated bootstraps, these mean differences of €2.99 (95% CI −€0.05 to €5.76, *p* = 0.054) for WTA and €2.81 per hour for WTP (95% CI −€0.08 to €5.71, *p* = 0.055) were almost significant. The lower value placed on one hour of care for those living separately from the care recipient is consistent with the lower levels of impact on quality of life, loneliness and total time spent caring also seen for the living separately group.

Another aspect of the value of caring is the travel costs associated with caring, particularly where carers live separately from the person they are caring for. Carers who live separately from the person they care for have much lower mean hours of care per week but may have more travel time costs and expenses. In our survey, on average, carers spent 3.31 h every week on care-related travel. This increased to 3.45 h for people living separately from the person they cared for. This difference was not significant. Our survey also indicates that only for carers who do not live with the person they care for, the mean cost of a return journey for carers to visit the person they support is €11.29, with a mean of 3.13 trips per week. We added travel expenses, plus travel time valued using the WTA hourly rate to the overall value of caring. This means that the overall weekly value of caring is €1441 using the WTA approach or €1164 using the WTP approach. Living with the person being cared for is associated with significantly increased costs; overall, using the WTA approach, the mean weekly value of caring would be €2223 compared with €794 for carers who live separately. These figures using the WTP methodology are €1758 and €660, respectively. After 1000 bias-corrected and accelerated bootstraps, these mean weekly differences of €1438 (95% CI €1070 to €1815, *p* = 0.001) for WTA and €1098 for WTP (95% CI €772 to €1414, *p* = 0.001) remained significant.

Extrapolated across an entire year, the average value of caring hours ranges between €74,932 and €60,904 depending on whether the WTA or WTP valuations of caring time are used. Costs for carers living with the care recipient are significantly greater, e.g., using the WTA approach, €116,084 versus €41,301 for carers living independently from the care recipient; after 1000 bias-corrected and accelerated bootstraps, there is a significant mean difference of €74,782 (95% CI €55,004 to €94,214, *p* = 0.001).

The monetary value of hours of caring represents only a part of the true value of care. In addition, there are also adverse impacts on quality of life. For illustrative purposes, we have conservatively valued each year of perfect quality of life at €30,000, in line with values seen in health economic studies in the UK and Spain [33,34]. If we include these impacts, then the total average annual economic impact of caring would increase to €84,466 (using the WTA value of carer hours). Annual costs for carers living with or without the care recipient would be €125,412 and €50,465, respectively.

## 4. Discussion

To our knowledge, this is the first multi-country survey looking at the economic value of informal care for all mental health conditions. Our survey of more than 700 carers highlights the tremendous and too often hidden multi-dimensional impacts and value of caregiving. Potentially, without the input of these (mainly close family) carers, undoubtedly some of this support would need to be provided instead by health and social care systems. A lack of support for carers is also likely to increase their chances of work cut back, complete withdrawal and premature retirement from the labour market, reducing national productivity and potentially impoverishing carers [35]. In short, informal carers are fundamental to the functioning of health and social care systems and the wider economy; it is critical therefore to invest in measures to support these caregivers and identify potential risk factors that might lead to a breakdown in caregiving support. We have highlighted that the average informal caring week, at more than 43 h, is longer than the typical working week, and that this is significantly greater at more than 65 h per week for carers who live with the person that they care for, compared to carers who live separately to the people they support.

### 4.1. Comparisons with Wider Literature

Our analysis is consistent with that seen in other studies that have looked at time spent caring, although most of those have focused on a subset of conditions rather than all conditions. Recent analysis from a convenience sample survey in Australia looking at time spent caring by family carers for a range of mental health conditions had similar findings to this study; they estimate that primary carers provide on average approximately 36 h of care per week, with 38% caring for 40 or more hours per week [3,21,22]. In contrast, a cross-sectional survey in Austria reported very low weekly hours of care of only 2.82 h per week, but this survey only interviewed people living with mental health conditions, rather than their carers [36]. In the Basque country, in Spain, a convenience sampling survey of more than 200 carers supporting people with eating disorders, depression and schizophrenia, respectively, reported that 44%, 68% and 33% of carers were in contact with the person they support for more than 35 h per week [37]. Another Spanish study drawing on survey data for people with disabilities reported between 31 and 70 h per week of informal care for people with moderate to severe schizophrenia [38]. Caregivers of people with schizophrenia in seven countries (Australia, Czech Republic, France, Italy, Russian Federation, Spain and Turkey) were also asked about caring hours; in Italy, the median number of hours of care per week was 53 compared to 19 in Spain [39].

We have highlighted the detrimental effects on carer quality of life, as well as much higher levels of loneliness and noted earlier that impacts on quality of life have been reported in the wider carer literature. These detrimental effects are likely to reflect the complexity of being a carer, with 46% trying to juggle both employment and caring, which can leave them vulnerable to both physical and mental health adverse impacts. Moreover, carers may be supporting someone with multiple mental health conditions, adding to the complexity of their needs. We have also seen that carers who reduce work have, on average, reduced paid employment by 19 h per week, which may help explain why more than half are worried about their finances. Carers also have concerns about their own future; with a mean age of over 53, many carers already have or are approaching retirement age, with one-third indicating that they have or may to postpone their own retirement because of their financial situation.

We found a much higher mean level of loneliness of 5.88 measured using the UCLA-3 than seen in other general population surveys in high-income settings—for example, a general population survey using the UCLA-3 of more than 10,500 adults in Finland, Poland and Spain reported mean scores of 3.51, 3.79, and 3.74, respectively [40]. Our UCLA-3 scores are also significantly higher than those seen in the general adult population over the age of 50 in England, measured in the English Longitudinal Survey of Ageing, where the latest mean loneliness score was 4.0 [41]. We ran 1000 bootstraps of a one-sample t-test compared against all of these mean scores; in all cases, our survey participants have significantly higher levels of loneliness (*p* = 0.001).

Asking carers to value their own caring opportunity costs rather than use the replacement cost conventions using hourly wage rates for professional carers should provide a better estimate of the economic value of care as it reflects carers own lived experiences of caring. We have estimated that the mean weekly value of informal care can be as much as €1441 when asking carers to put a monetary value on the costs of their caring time. Because of the greater number of caring hours provided by carers living with the person they care for, the value of caring time for these carers can be much greater at €2223 per week. The mean annual value of informal care, including impacts on quality of life, could be as high as €84,466 (using the WTA value of carer hours), ranging between €125,412 and €50,465 depending on carer living arrangements.

To our knowledge, there are very few recent comparable estimates of these costs. An analysis of survey data in 2013 on the informal care costs for schizophrenia in Spain, valued using the replacement cost approach, estimated costs to be between €855 and €1417 per week [38]. In an Australian survey, the mean annual value of time spent supporting someone with a mental health condition (valued using a replacement cost approach) is €77,351 [3,21]. This is similar to our estimate, as it does not include quality of life impacts.

There are also examples of studies that report on aspects of the costs of caring, but do not report the overall costs of care. Using data from the 2012 US National Health and Wellness Survey, researchers found that carers of people with schizophrenia had higher levels of absenteeism from work and poor performance at work compared to other carers and non-carers, but the analysis did not measure total hours of care provided [42]. In another study, analysis of the experience of caring for someone with schizophrenia and caring for other (physical health) client groups was compared across five countries [43]. It found that family caregivers for people with schizophrenia were more likely to take time off work and had significantly greater contact with primary and secondary services than other carers. A similar study in Sweden also reported that parental carers of people with schizophrenia were much more likely to need specialist care for their own mental health, and more likely to lose employment, compared to parental carers of people with rheumatoid arthritis, multiple sclerosis and epilepsy [44]. In summary, however, the existing evidence base is limited, using very different methodologies, making comparisons difficult.

### 4.2. Policy Implications

Potentially there are important health, well-being and economic gains to be had from providing more support to carers, and in particular providing support for carers who live with the person they care for, as they appear to provide more intensive care, as well as supporting carers supporting people with more complex multiple mental health problems regardless of living arrangements. Respite from care is one key consideration; however, we are unaware of any evaluation in the literature on the impacts or cost effectiveness of respite on the well-being of carers for people with mental health conditions (other than dementia), and this needs to be evaluated. Peer support may also play a vital role; caring can be daunting and having support from individuals who have or are going through the same process can be helpful. Both greater access to respite care and more peer support may also help to reduce the sense of loneliness that is felt by many carers. Measures that can help maintain carers social capital through connections with their local communities may also help to safeguard their mental health [45]. Governments’ may also wish to reflect on whether what typically is very limited financial support for carers of working age should be increased.

### 4.3. Limitations

While our analysis provides important insights on the impacts and value of care that can help facilitate policy and practice change, there are limitations in our methodology so these findings must be treated cautiously. This is a cross-sectional survey, so we cannot make any inferences on causality. Longitudinal studies that follow up carers over time are needed to understand how caring experiences change. These surveys can also monitor receipt of interventions to consider whether they have an impact on the challenges associated with caregiving. The scale of surveys also needs to increase, with a focus on approaches that can obtain a fully representative sample of the caregiver population.

Recruitment to our survey was through convenience sampling, mainly dependent on awareness-raising actions by the EUFAMI through social media and its membership base. This means that our carer sample may not be representative of all carers for people with mental health conditions, although the number of respondents in our selected countries was similar to that of an earlier survey commissioned by the EUFAMI where similar recruitment methods were used: 657 carers versus 712 carers in our survey [2]. Many of our respondents are likely to be members of family associations and thus be more informed and have access to more support than carers who are not members of family associations. Most carers in our survey (64%) were also ‘experienced’ carers who had been supporting someone with a mental health condition for more than 3 years. Our reliance on an online survey also means that carers without these technologies, as well as those who are reluctant to use these technologies, will also be missing. All of these factors might suggest that experiences and value of caring obtained from our survey might be conservative compared to carers who are not connected to support associations, and/or those with little use of the internet and who therefore may be less aware of formal supports that may be available.

Larger representative surveys (or replication surveys within countries) using probability-based sampling methods would also allow more definitive statements to be made about caring experiences at an individual country level, something that was not possible because of the relatively small sample size in this study. Our survey included countries from at least four distinct welfare systems, where the generosity of social welfare benefits vary, which could impact on findings. However, we did not find any difference in caring experiences linked to welfare benefits across countries, as only 30 (7%) of 429 responding carers indicated that they received any financial support from their governments for being a carer, whilst only 63 (14%) indicated they received regular support from paid carers.

Looking at our estimate of costs, these do not account for levels of informal care that would be provided in all households even without health problems. However, we believe our estimate of costs may still be conservative. Loneliness, for example, can be associated with substantial economic costs, due to the increased risk of both physical and mental health problems [46]. Even though we identified high levels of loneliness in carers, we are not able to put a monetary value on these outcomes, nor can we assume that quality of life captures the impacts of loneliness, or even the stigma that may be associated with caring. We have also not included any costs associated with any increased use of health care services by carers; these, again, may not be captured by changes in quality of life. We also have not attached any monetary value to lost long-term career opportunities. More research is also needed on the economic value and impact of caring for people with less common mental health problems. For instance, our survey suggests very adverse outcomes for carers of people with eating disorders, but the numbers are too small to draw any conclusions.

## 5. Conclusions

Few studies have looked at the economic value provided by informal carers in supporting people living with mental health conditions. Our survey found that, on average, they contribute more than 43 h of care per week, rising to 65 h for carers living with care recipients. This vital support has tremendous economic value, an average of up to €84,000 per annum. Carers are also at risk of poor quality of life, higher levels of loneliness, and financial distress.

Potentially therefore there is a good case for investing in measures to support informal carers, protecting their physical, mental and financial health. If carers are unable or unwilling to provide support, our analysis indicates that substantial additional costs may fall on health and social care services to replace this care. Longitudinal studies are, however, needed to better quantify the long-term impacts of informal care, as well as the effectiveness of measures to support carers.

It is perhaps especially salient that the high economic value of informal care that we have observed refers to the period immediately before the COVID-19 pandemic. The pandemic has further increased reliance on informal family care, often to the detriment of carer physical and mental health [47]; this is very likely to also have exacerbated the adverse economic impacts of caring on carers, strengthening the case for action to support carers further.

## Figures and Tables

**Table 1:** Carer characteristics.

	Overall (712)	%	Lives Together (300)	%	Lives Separately (371)	%
Gender and age						
Male	124	17%	61	20%	60	16%
Mean age (SD)	53.4 (13.7)		52.4 (13.0)		54.7 (14.0)	
18–24 years	24	3%	10	3%	13	4%
25–64 years	494	69%	227	76%	252	68%
65+ years	135	19%	47	16%	88	24%
Age not reported (NR)	59	8%	16	5%	18	5%
Civil status						
Single/Separated/Widowed	259	37%	105	35%	140	39%
Married/Civil partnership/Co-habiting	368	52%	160	53%	194	52%
Other	51	7%	23	8%	26	7%
NR	34	5%	12	4%	8	2%
Labour force status						
Employed	325	46%	133	44%	178	48%
Retired	187	26%	68	23%	118	32%
Student	24	3%	8	3%	15	4%
Disabled/unemployed/homeworker	100	14%	62	21%	32	9%
NR	76	11%	29	10%	28	8%
Caring role						
Sole unpaid carer	331	47%	167	56%	132	36%
Primary unpaid carer but share responsibilities	174	24%	66	22%	105	28%
Caring responsibilities shared equally	81	11%	29	10%	52	14%
Secondary unpaid carer	34	5%	10	3%	22	6%
Other	91	13%	27	9%	60	16%
NR	1	0%	1	0%	0	0%
Length of time caring						
0–6 months	38	5%	10	3%	28	8%
7–12 months	17	2%	10	3%	6	2%
13–24 months	83	12%	31	10%	35	9%
25–36 months	56	8%	23	8%	28	8%
More than 3 years	453	64%	207	69%	246	66%
NR	65	9%	19	6%	28	8%
Person being cared for						
Partner/Spouse	73	10%	61	20%	12	3%
Son/Daughter	483	68%	202	67%	249	67%
Brother/Sister	55	8%	13	4%	41	11%
Parent	38	5%	10	3%	27	7%
Other relative, friend, other	63	9%	14	5%	42	11%

**Table 2:** Care recipient characteristics.

	Overall (712)	%	Living Together (300)	%	Living Separately (371)	%
Gender						
Male	395	56%	189	63%	196	53%
Female	254	36%	98	33%	147	40%
Other	16	2%	1	0%	13	4%
NR	47	7%	12	4%	15	4%
Age in years						
Mean age (SD)	37.4 (15.8)		36.8 (15.8)		38.1 (15.9)	
18–24	151	21%	71	23%	75	20%
25–64	449	63%	194	65%	248	67%
65+	54	8%	22	7%	32	9%
NR	58	8%	13	4%	16	4%
Mental health condition						
Mean number of conditions (SD)	1.54 (1.38)		1.62 (1.38)		1.64 (1.37)	
Psychosis	317	45%	133	44%	184	50%
Mood disorder	208	29%	91	30%	117	32%
Anxiety disorder	168	24%	80	27%	88	24%
Personality disorder	91	13%	36	12%	55	15%
Eating disorder	33	5%	17	6%	16	4%

**Table 3:** Impacts and value of caring.

Variable (SD)	No. Obs	Overall	No. Obs	Living Together	No. Obs	Living Separately
CarerQoL-7D (SD)	518	60.17 (21.38)	236	55.93 (22.43)	279	63.75 (19.92) *
Happiness (SD)	512	49.01 (23.26)	233	43.71 (22.89)	276	53.42 (22.76) *
UCLA-3 (SD)	520	5.88 (2.03)	241	6.56 (1.95)	279	5.30 (1.92) *
Financial Concerns						
Greatly concerned	235	57 (24%)	120	35 (12%)	115	22 (19%)
Moderate/quite a lot of concern	235	75 (32%)	120	43 (36%)	115	32 (28%)
Working Hours						
Reduced working hours	491	213 (43%)	227	108 (48%)	258	102 (39%)
Mean weekly hours reduced (SD)	213	19.49 (11.94)	108	21.05 (11.54)	102	18.00 (12.27)
Retirement Income						
Greatly concerned	231	76 (33%)	117	41 (35%)	114	35 (30%)
Moderate/quite a lot of concern	231	68 (29%)	117	39 (33%)	114	29 (25%)
Have or will delay retirement	233	84 (36%)	121	52 (43%)	110	32 (29%)

* Living separately versus living together *p* = 0.001.

**Table 4:** Factors associated with loneliness levels in informal carers.

Factors	Coefficient	Standard Error	*t*	*p* > *t* *	95% CI	
Carer age	−0.011	0.006	−2.050	**0.041**	−0.022	0.000
Carer gender	−0.086	0.158	−0.550	0.585	−0.396	0.223
Live separately	−0.419	0.161	−2.600	**0.009**	−0.735	−0.103
Perceived happiness	−0.018	0.004	−4.980	**0.000**	−0.026	−0.011
Carer role	−0.034	0.056	−0.620	0.537	−0.143	0.075
Hours of care	0.009	0.002	5.130	**0.000**	0.006	0.013
Quality of life scores	−0.029	0.004	−6.980	**0.000**	−0.037	−0.021
Constant	9.638	0.551	17.480	0.000	8.555	10.722

* Bold values indicate *p* < 0.05.

**Table 5:** Value of caring.

Caring Value	No. Obs	Overall	No. Obs	Living Together	No. Obs	Living Separately
Caring Time per Week						
Mean hours of care (SD)	563	43.43 (45.31)	249	65.42 (51.50)	311	25.71 (29.80) *
Travel hours (SD)	563	3.31 (4.69)	249	2.75 (6.00)	311	3.45 (4.36)
Caring Value						
WTA per caring hour (SD)	522	€28.69 (€17.90)	229	€30.44 (€18.71)	274	€27.46 (€17.24)
WTP per caring hour (SD)	522	€23.57 (€16.24)	229	€25.07 (€16.90)	274	€22.27 (€15.64)
Weekly value of caring (WTA) (SD)	522	€1440 (€2208)	229	€2232 (€2735)	271	€794 (€1360) *
Weekly value of caring (WTP) (SD)	522	€1164 (€1760)	229	€1758 (€2136)	271	€660 (€1161) *
Potential Annual Value of Caring per Carer						
WTA approach	522	€74,932 (€114,792)	229	€116,084 (€142,202)	274	€41,301 (€70,721) *
WTP approach	522	€60,904 (€91,886)	229	€91,770 (€111,550)	274	€34,960 (€60,905) *
WTA plus QoL impacts	522	€84,466 (€115,581)	229	€125,412 (€143,074)	271	€50,465 (€70,969) *
WTP plus QoL impacts	522	€70,543 (€92,610)	229	€101,879 (€112,660)	271	€44,199 (€60,978) *

* Living separately versus living together *p* = 0.001.

## Data Availability

Data presented in this survey are available on reasonable request from the corresponding author. Full data are not publicly available due to privacy restrictions.

## Supplementary Materials

The following supporting information can be downloaded at: https://www.mdpi.com/article/10.3390/ijerph19052858/s1, English version of carer survey.
