# Supplementary material for: Understanding the Economic Value and Impacts on Informal Carers of People Living with Mental Health Conditions
Source: Int J Environ Res Public Health. 2022 Mar 1;19(5):2858. doi: 10.3390/ijerph19052858 (PMC8910204; doi:10.3390/ijerph19052858)
Supplement: Supplementary file 1 [file ijerph-19-02858-s001.zip › ijerph-1561599-supplementary.pdf]

English Version of Carer Survey

Start of Block: Consent

Q195
INFORMATION SHEET

We are inviting you to participate in this online survey of your experiences of caring for a person with poor mental health. The survey has been commissioned and is being conducted by EUFAMI, the European Federation of Associations of Families of People with Mental Illness, Leuven, Belgium. The London School of Economics and Political Science, in London, UK are working with EUFAMI to analyse and summarise the findings.

The results will be used to help estimate all of the value to society of informal caring for people with mental health needs to inform national and local policymaking, strengthen public awareness of the contribution of informal carers and advocate for greater recognition of mental health carers and their support needs.

All information you provide will be treated in a confidential manner and will be kept securely by EUFAMI for up to 10 years. You do not have to finish the survey in one go; your responses are automatically saved and you can come back to complete within a week. You can also go back and change any of your answers at any time until you submit the questionnaire, but once this is submitted it will no longer be possible to change your responses.

More information on this survey as well as an option to print and post the survey instead are available on the EUFAMI website http://eufami.org/economic-case-survey/ . If you have any questions or concerns please feel free to contact Margaret Walker, Executive Director at EUFAMI. Email: executive.director@eufami.org Telephone: + 32 468 17 71 48

Q1

To be eligible to participate in the survey you should:
1) **Be 18 years or older;**
2) **Care for someone aged 18 years or older with at least one severe mental health condition** (other than dementia and learning difficulties). You do not have to be their main carer or live at the same address.
3) **Not be employed to provide your caring role** (i.e. formally paid a wage for caring). You can still participate in the study if you receive a government benefit such as the Carer's

Allowance.

This study has received ethical clearance from the London School of Economics and Political Science. There are no specific risks to your health and wellbeing associated with participation in this survey. You can change your responses until you have clicked the “Submit” button at the end of the survey. Once submitted your responses cannot be changed.

Statement of informed consent

If you select **I AGREE** this means that you have read this information sheet, understand the purpose and nature of the survey and are participating voluntarily. If you do not wish to participate in the research study, please decline participation by selecting “**I DISAGREE**”.

- I AGREE (1)
- I DISAGREE (2)

Skip To: End of Survey If To be eligible to participate in the survey you should: 1) Be 18 years or older; 2) Care for som... = I DISAGREE

End of Block: Consent

Start of Block: Country of residence

X→

Q103 In which country do you currently reside?

- ◯ England (1)
- ◯ Northern Ireland (2)
- ◯ Scotland (3)
- ◯ Wales (4)
- ◯ Ireland (5)
- ◯ Afghanistan (6)
- ◯ Albania (7) ……….
- ◯ Zimbabwe (199)

End of Block: Country of residence

Start of Block: about carer_1

Q252 Which of the following best describes your role as a carer? (Here we are referring to unpaid informal care only)

- ◯ I am the only unpaid caregiver (1)
- ◯ I share caring responsibilities with others, but I am the main unpaid caregiver (2)
- ◯ I share caring responsibilities equally with other unpaid carers (3)
- ◯ I share caring responsibilities, but someone else is the main unpaid caregiver (4)
- ◯ Other (5)

Q245 The person I care for is my

- ◯ Partner / Spouse (1)
- ◯ Son / Daughter (2)
- ◯ Brother / Sister (3)
- ◯ Parent (4)
- ◯ Other Relative (5)
- ◯ Friend (6)
- ◯ Other (7)

Q237 What is your gender?

- ◯ Male (1)
- ◯ Female (2)
- ◯ Other (4)

Q241 What is your age?
Age in Years (1)

- 10 (1)
- 11 (2)
- 12 (3)
- 13 (4)
- 14 (5)
- 15 (6)
- 16 (7)
- 17 (8)
- 18 (9)
- 19 (10)
- 20 (11)
- 21 (12)
- 22 (13)
- 23 (14)
- 24 (15)
- 25 (16)
- 26 (17)
- 27 (18)
- 28 (19)
- 29 (20)

- ○ 30 (21)
- ○ 31 (22)
- ○ 32 (23)
- ○ 33 (24)
- ○ 34 (25)
- ○ 35 (26)
- ○ 36 (27)
- ○ 37 (28)
- ○ 38 (29)
- ○ 39 (30)
- ○ 40 (31)
- ○ 41 (32)
- ○ 42 (33)
- ○ 43 (34)
- ○ 44 (35)
- ○ 45 (36)
- ○ 46 (37)
- ○ 47 (38)
- ○ 48 (39)
- ○ 49 (40)
- ○ 50 (41)

- ○ 51 (42)
- ○ 52 (43)
- ○ 53 (44)
- ○ 54 (45)
- ○ 55 (46)
- ○ 56 (47)
- ○ 57 (48)
- ○ 58 (49)
- ○ 59 (50)
- ○ 60 (51)
- ○ 61 (52)
- ○ 62 (53)
- ○ 63 (54)
- ○ 64 (55)
- ○ 65 (56)
- ○ 66 (57)
- ○ 67 (58)
- ○ 68 (59)
- ○ 69 (60)
- ○ 70 (61)
- ○ 71 (62)

- 72 (63)
- 73 (64)
- 74 (65)
- 75 (66)
- 76 (67)
- 77 (68)
- 78 (69)
- 79 (70)
- 80 (71)
- 81 (72)
- 82 (73)
- 83 (74)
- 84 (75)
- 85 (76)
- 86 (77)
- 87 (78)
- 88 (79)
- 89 (80)
- 90 (81)
- 91 (82)
- 92 (83)

- 93 (84)
- 94 (85)
- 95 (86)
- 96 (87)
- 97 (88)
- 98 (89)
- 99 (90)
- 100 (91)
- 101 (92)
- 102 (93)
- 103 (94)
- 104 (95)
- 105 (96)
- 106 (97)
- 107 (98)
- 108 (99)
- 109 (100)

Q242 What is your marital status

- ○ Single (1)
- ○ Married/Same sex civil partnership/Co-habiting (2)
- ○ Separated/Divorced (3)
- ○ Widowed/surviving partner from a civil partnership (6)
- ○ Other (please state) (4)
- ○ Prefer not to say (7)

Q109 Which of these catagories best describes your current status?

- ○ Employed (6)
- ○ Retired (1)
- ○ Student (2)
- ○ Permanently sick / disabled (3)
- ○ Looking for work (7)
- ○ Looking after home / family (4)
- ○ None of the above (5)

End of Block: about carer_1

Start of Block: brief info on person cared for

Q246 The person i care for is

- ○ Male (1)
- ○ Female (2)
- ○ Other (8)

Q248 How old is the person you care for?
Age in years (4)

- ○ 18 (1)
- ○ 19 (2)
- ○ 20 (3)
- ○ 21 (4)
- ○ 22 (5)
- ○ 23 (6)
- ○ 24 (7)
- ○ 25 (8)
- ○ 26 (9)
- ○ 27 (10)
- ○ 28 (11)
- ○ 29 (12)
- ○ 30 (13)
- ○ 31 (14)
- ○ 32 (15)
- ○ 33 (16)
- ○ 34 (17)
- ○ 35 (18)
- ○ 36 (19)
- ○ 37 (20)

- 38 (21)
- 39 (22)
- 40 (23)
- 41 (24)
- 42 (25)
- 43 (26)
- 44 (27)
- 45 (28)
- 46 (29)
- 47 (30)
- 48 (31)
- 49 (32)
- 50 (33)
- 51 (34)
- 52 (35)
- 53 (36)
- 54 (37)
- 55 (38)
- 56 (39)
- 57 (40)
- 58 (41)

- ○ 59 (42)
- ○ 60 (43)
- ○ 61 (44)
- ○ 62 (45)
- ○ 63 (46)
- ○ 64 (47)
- ○ 65 (48)
- ○ 66 (49)
- ○ 67 (50)
- ○ 68 (51)
- ○ 69 (52)
- ○ 70 (53)
- ○ 71 (54)
- ○ 72 (55)
- ○ 73 (56)
- ○ 74 (57)
- ○ 75 (58)
- ○ 76 (59)
- ○ 77 (60)
- ○ 78 (61)
- ○ 79 (62)

- ○ 80 (63)
- ○ 81 (64)
- ○ 82 (65)
- ○ 83 (66)
- ○ 84 (67)
- ○ 85 (68)
- ○ 86 (69)
- ○ 87 (70)
- ○ 88 (71)
- ○ 89 (72)
- ○ 90 (73)
- ○ 91 (74)
- ○ 92 (75)
- ○ 93 (76)
- ○ 94 (77)
- ○ 95 (78)
- ○ 96 (79)
- ○ 97 (80)
- ○ 98 (81)
- ○ 99 (82)
- ○ 100 (83)

○ 101 (84)

○ 102 (85)

○ 103 (86)

○ 104 (87)

○ 105 (88)

○ 106 (89)

○ 107 (90)

○ 108 (91)

○ 109 (92)

Q250 Does the person you care for live with you?

○ Yes (1)

○ No (2)

Q251 How long have you been caring because of his/her mental health needs?

- ○ 0 - 3 months (1)
- ○ 4 -6 months (2)
- ○ 7 - 12 months (3)
- ○ up to 18 months (4)
- ○ up to 2 years (7)
- ○ up to 3 years (8)
- ○ More than 3 years (9)

Q253 Has the person you care for been diagnosed with one or more of the following mental health conditions? Select **ALL** that apply

- ☐ Schizophrenia (1)
- ☐ Schizoaffective Disorder (2)
- ☐ Other Psychosis (3)
- ☐ Bipolar Disorder (Manic Depression) (4)
- ☐ Depression Major Depressive Disorder) (5)
- ☐ General Anxiety Disorder (6)
- ☐ Panic Disorder (with or without agrophobia) (7)
- ☐ OCD (Obsessive Compulsive Disorder) (8)
- ☐ PTSD (Post Traumatic Stress Disorder) (9)
- ☐ Social Phobia (10)
- ☐ Other Specific Phobia (11)
- ☐ Borderline Personality Disorder (BPD) (12)
- ☐ Other Personality Disorder (13)
- ☐ Anorexia or Bulimia (Eating Disorder) (14)
- ☐ Other (15)
- ☐ Dont know / Not had a diagnosis (16)

Display This Question:

If If Has the person you care for been diagnosed with one or more of the following mental health conditions? Select ALL that apply q://QID13/SelectedChoicesCount Is Greater Than 1

Q255 What do you consider to be his/her main mental health condition?

- Schizophrenia (1)
- Schizoaffective Disorder (2)
- Other Psychosis (3)
- Bipolar Disorder (Manic Depression) (4)
- Depression (Major Depressive Disorder) (5)
- General Anxiety Disorder (6)
- Panic Disorder (with or without agrophobia) (7)
- OCD (Obsessive Compulsive Disorder) (8)
- PTSD (Post Traumatic Stress Disorde) (9)
- Social Phobia (10)
- Other Specific Phobia (11)
- Borderline Personality Disorder (12)
- Other Personality Disorder (13)
- Anorexia or Bulimia (Eating Disorder) (14)
- Other (15)

End of Block: brief info on person cared for

Start of Block: Section B1: Overall time spent caring

Q26 In a typical week, how many hours in total do you spend providing care? This could range between 0 and 168 hours per week

	0 17 34 50 67 84 101 118 134 151 168
Total hours of care provided per week ()	

End of Block: Section B1: Overall time spent caring

Start of Block: ucla-3

Q124 We would like to know more about the impacts of caring on your social situation. Please select the choices that best reflect your situation

	Hardly ever (1)	Some of the time (2)	Often (3)
How often do you feel that you lack companionship? (1)	○	○	○
How often do you feel left out? (2)	○	○	○
How often do you feel isolated from others? (3)	○	○	○

End of Block: ucla-3

Start of Block: Section C: Understanding the value of caring

Q32 We would like to form an impression of your caregiving situation. Please choose between 'no', 'some' and 'a lot of' for each of the following descriptions

	No (1)	Some (2)	A lot of (3)
I have no/some/a lot of fulfilment from carrying out my care tasks (1)	○	○	○
I have no/some/a lot of relational problems with the care receiver (e.g. he/she is very demanding or he/she behaves differently; we have communication problems. (2)	○	○	○
I have no/some/a lot of problems with my own mental health (e.g. stress, fear, gloominess, depression, concern about the future) (3)	○	○	○
I have no/ some/ a lot of problems combining my care tasks with my daily activities (e.g., household activities, work, study, family and leisure activities) (7)	○	○	○
I have no/some/a lot of financial problems because of my care tasks (4)	○	○	○
I have no/some/a lot of support with carrying out my care taks, when I need it (e.g. from family, friends, neighbours, acquantances) (5)	○	○	○
have no/some/a lot of problems with my	○	○	○

own physical health (e.g. more often sick, tiredness, physical stress) (6)	

Q33 How happy do you feel at the moment? *Please indicate on the scale how happy you feel at the moment where 0 indicates completely unhappy and 100 indicates completely happy*

	Completely unhappy Completely happy<br>0 10 20 30 40 50 60 70 80 90 100
1 ()	

Q34 Imagine that the person you are caring for needs one extra hour of informal care per week and that the government will pay you for providing this extra hour of informal care. Which net amount from the government would you minimally want to receive in order to provide one extra hour of care?
Net amount you would need to receive to provide an extra hour of care (1)

- ○ Less than 5 pounds (1)
- ○ 5 - 10 pounds (2)
- ○ 11 - 15 pounds (3)
- ○ 16 - 20 pounds (4)
- ○ 21 - 25 pounds (5)
- ○ 26 - 30 pounds (6)
- ○ 31 - 35 pounds (7)
- ○ 36 - 40 pounds (8)
- ○ 41 - 45 pounds (9)
- ○ 46 - 50 pounds (10)
- ○ More than 50 pounds (11)

Q35 Imagine that you get the opportunity to give up one hour of informal care per week. Somebody else has offered to provide this hour of informal care instead, so the total number of hours of care will not change. How much would you personally be willing to pay to them so that you can provide one less hour of care per week?
Amount you would be willing to pay for someone else to provide care (1)

- ○ Less than 5 pounds (1)
- ○ 5 - 10 pounds (2)
- ○ 11 - 15 pounds (3)
- ○ 16 - 20 pounds (4)
- ○ 21 - 25 pounds (5)
- ○ 26 - 30 pounds (6)
- ○ 31 - 35 pounds (7)
- ○ 36 - 40 pounds (8)
- ○ 41 - 45 pounds (9)
- ○ 46 - 50 pounds (10)
- ○ More than 50 pounds (11)

End of Block: Section C: Understanding the value of caring

Start of Block: Impacts of caring on employment, education, leisure and family life

Q125 We would like to know about the impacts of caring on employment, education, volunteering and leisure time. Please select the options that best reflect your situation.

	Yes (1)	No (3)
I have reduced time spent working (1)	○	○
I have reduced time volunteering (2)	○	○
I have reduced time in education (4)	○	○
I have reduced my leisure time (5)	○	○

Display This Question:

If We would like to know about the impacts of caring on employment, education, volunteering and leis... != I have reduced time spent working [No]

Q37 How many hours of work per week did you give up?
Number of hours per week (7)

- ○ 1 (1)
- ○ 2 (2)
- ○ 3 (3)
- ○ 4 (4)
- ○ 5 (5)
- ○ 6 (6)
- ○ 7 (7)
- ○ 8 (8)
- ○ 9 (9)
- ○ 10 (10)
- ○ 11 (11)
- ○ 12 (12)
- ○ 13 (13)
- ○ 14 (14)
- ○ 15 (15)
- ○ 16 (16)
- ○ 17 (17)
- ○ 18 (18)
- ○ 19 (19)
- ○ 20 (20)

○ 21 (21)

○ 22 (22)

○ 23 (23)

○ 24 (24)

○ 25 (25)

○ 26 (26)

○ 27 (27)

○ 28 (28)

○ 29 (29)

○ 30 (30)

○ 31 (31)

○ 32 (32)

○ 33 (33)

○ 34 (34)

○ 35 (35)

○ 36+ (36)

Display This Question:

If We would like to know about the impacts of caring on employment, education, volunteering and leis... != I have reduced time volunteering [No]

Q39 How many hours of volunteering per week did you give up?
Number of hours per week (4)

- ○ 1 (1)
- ○ 2 (2)
- ○ 3 (3)
- ○ 4 (4)
- ○ 5 (5)
- ○ 6 (6)
- ○ 7 (7)
- ○ 8 (8)
- ○ 9 (9)
- ○ 10 (10)
- ○ 11 (11)
- ○ 12 (12)
- ○ 13 (13)
- ○ 14 (14)
- ○ 15 (15)
- ○ 16 (16)
- ○ 17 (17)
- ○ 18 (18)
- ○ 19 (19)
- ○ 20 (20)

- 21 (21)
- 22 (22)
- 23 (23)
- 24 (24)
- 25 (25)
- 26 (26)
- 27 (27)
- 28 (28)
- 29 (29)
- 30 (30)
- 31 (31)
- 32 (32)
- 33 (33)
- 34 (34)
- 35 (35)
- 36+ (36)

Display This Question:

If We would like to know about the impacts of caring on employment, education, volunteering and leis... != I have reduced time in education [No]

Q41 How many hours of education per week did you give up?
Number of hours per week (4)

- ○ 1 (1)
- ○ 2 (2)
- ○ 3 (3)
- ○ 4 (4)
- ○ 5 (5)
- ○ 6 (6)
- ○ 7 (7)
- ○ 8 (8)
- ○ 9 (9)
- ○ 10 (10)
- ○ 11 (11)
- ○ 12 (12)
- ○ 13 (13)
- ○ 14 (14)
- ○ 15 (15)
- ○ 16 (16)
- ○ 17 (17)
- ○ 18 (18)
- ○ 19 (19)
- ○ 20 (20)

○ 21 (21)

○ 22 (22)

○ 23 (23)

○ 24 (24)

○ 25 (25)

○ 26 (26)

○ 27 (27)

○ 28 (28)

○ 29 (29)

○ 30 (30)

○ 31 (31)

○ 32 (32)

○ 33 (33)

○ 34 (34)

○ 35 (35)

○ 36+ (36)

Display This Question:

If We would like to know about the impacts of caring on employment, education, volunteering and leis... != I have reduced my leisure time [No]

Q43 How many hours of leisure time per week did you give up?
Number of hours per week (4)

- ○ 1 (1)
- ○ 2 (2)
- ○ 3 (3)
- ○ 4 (4)
- ○ 5 (5)
- ○ 6 (6)
- ○ 7 (7)
- ○ 8 (8)
- ○ 9 (9)
- ○ 10 (10)
- ○ 11 (11)
- ○ 12 (12)
- ○ 13 (13)
- ○ 14 (14)
- ○ 15 (15)
- ○ 16 (16)
- ○ 17 (17)
- ○ 18 (18)
- ○ 19 (19)
- ○ 20 (20)

- 21 (21)
- 22 (22)
- 23 (23)
- 24 (24)
- 25 (25)
- 26 (26)
- 27 (27)
- 28 (28)
- 29 (29)
- 30 (30)
- 31 (31)
- 32 (32)
- 33 (33)
- 34 (34)
- 35 (35)
- 36+ (36)

Q44 Suppose you did not have to provide informal care anymore. How would you spend this time?

	Yes (1)	No (2)
More work (1)	○	○
More volunteering (6)	○	○
More education (7)	○	○
More leisure (8)	○	○

Display This Question:

If Suppose you did not have to provide informal care anymore. How would you spend this time? = More work [Yes]

Q45 How many extra hours of work per week would you like to do?
Number of hours per week (4)

- ○ 1 (1)
- ○ 2 (2)
- ○ 3 (3)
- ○ 4 (4)
- ○ 5 (5)
- ○ 6 (6)
- ○ 7 (7)
- ○ 8 (8)
- ○ 9 (9)
- ○ 10 (10)
- ○ 11 (11)
- ○ 12 (12)
- ○ 13 (13)
- ○ 14 (14)
- ○ 15 (15)
- ○ 16 (16)
- ○ 17 (17)
- ○ 18 (18)
- ○ 19 (19)
- ○ 20 (20)

○ 21 (21)

○ 22 (22)

○ 23 (23)

○ 24 (24)

○ 25 (25)

○ 26 (26)

○ 27 (27)

○ 28 (28)

○ 29 (29)

○ 30 (30)

○ 31 (31)

○ 32 (32)

○ 33 (33)

○ 34 (34)

○ 35 (35)

○ 36+ (36)

Display This Question:

If Suppose you did not have to provide informal care anymore. How would you spend this time? = More volunteering [Yes]

Q47 How many extra hours of volunteering per week would you like to do?
Number of hours per week (4)

- ○ 1 (1)
- ○ 2 (2)
- ○ 3 (3)
- ○ 4 (4)
- ○ 5 (5)
- ○ 6 (6)
- ○ 7 (7)
- ○ 8 (8)
- ○ 9 (9)
- ○ 10 (10)
- ○ 11 (11)
- ○ 12 (12)
- ○ 13 (13)
- ○ 14 (14)
- ○ 15 (15)
- ○ 16 (16)
- ○ 17 (17)
- ○ 18 (18)
- ○ 19 (19)
- ○ 20 (20)

- 21 (21)
- 22 (22)
- 23 (23)
- 24 (24)
- 25 (25)
- 26 (26)
- 27 (27)
- 28 (28)
- 29 (29)
- 30 (30)
- 31 (31)
- 32 (32)
- 33 (33)
- 34 (34)
- 35 (35)
- 36+ (36)

Display This Question:

If Suppose you did not have to provide informal care anymore. How would you spend this time? = More education [Yes]

Q46 How many extra hours of education per week would you like to do?
Number of hours per week (4)

- 1 (1)
- 2 (2)
- 3 (3)
- 4 (4)
- 5 (5)
- 6 (6)
- 7 (7)
- 8 (8)
- 9 (9)
- 10 (10)
- 11 (11)
- 12 (12)
- 13 (13)
- 14 (14)
- 15 (15)
- 16 (16)
- 17 (17)
- 18 (18)
- 19 (19)
- 20 (20)

- 21 (21)
- 22 (22)
- 23 (23)
- 24 (24)
- 25 (25)
- 26 (26)
- 27 (27)
- 28 (28)
- 29 (29)
- 30 (30)
- 31 (31)
- 32 (32)
- 33 (33)
- 34 (34)
- 35 (35)
- 36+ (36)

Display This Question:

If Suppose you did not have to provide informal care anymore. How would you spend this time? = More leisure [Yes]

Q48 How many extra hours of leisure per week would you like to have?
Number of hours per week (9)

- ○ 1 (1)
- ○ 2 (2)
- ○ 3 (3)
- ○ 4 (4)
- ○ 5 (5)
- ○ 6 (6)
- ○ 7 (7)
- ○ 8 (8)
- ○ 9 (9)
- ○ 10 (10)
- ○ 11 (11)
- ○ 12 (12)
- ○ 13 (13)
- ○ 14 (14)
- ○ 15 (15)
- ○ 16 (16)
- ○ 17 (17)
- ○ 18 (18)
- ○ 19 (19)
- ○ 20 (20)

- 21 (21)
- 22 (22)
- 23 (23)
- 24 (24)
- 25 (25)
- 26 (26)
- 27 (27)
- 28 (28)
- 29 (29)
- 30 (30)
- 31 (31)
- 32 (32)
- 33 (33)
- 34 (34)
- 35 (35)
- 36+ (36)

End of Block: Impacts of caring on employment, education, leisure and family life

---

Start of Block: Impacts on carer health_rev

Q262 Please indicate if you have been in contact with any of the following services because of your health in the last 3 months

	Yes (1)	No (2)
GP (1)	○	○
GP Practice Nurse (2)	○	○
Psychologist (3)	○	○
Phsyiotherapist (4)	○	○
Occupational Therapist (5)	○	○
Social Worker (6)	○	○
Community Mental Health Team (7)	○	○
Complementary / Alternative Medicine (e.g. Acupuncture, Mindfulness, Yoga etc) (8)	○	○
Other therapist (9)	○	○
Visits to hospital not involving an overnight stay (11)	○	○
Visits to hospital involving an overnight stay (12)	○	○

Display This Question:

If Please indicate if you have been in contact with any of the following services because of your he... = GP [Yes]

Q263 Please indicate the number of visits to your GP in the last 3 months
Number of visits (1)

- 0 (1)
- 1 (2)
- 2 (3)
- 3 (4)
- 4 (5)
- 5 (6)
- 6 (7)
- 7 (8)
- 8 (9)
- 9 (10)
- 10 (11)
- 11 (12)
- 12 (13)
- 13 (14)
- 14 (15)
- 15 (16)
- 16 (17)
- 17 (18)
- 18 (19)
- 19 (20)

○ 20 (21)

○ 21 (22)

○ 22 (23)

○ 23 (24)

○ 24 (25)

○ 25 (26)

○ 26 (27)

○ 27 (28)

○ 28 (29)

○ 29 (30)

○ 30 (31)

○ 31 (32)

○ 32 (33)

○ 33 (34)

○ 34 (35)

○ 35 (36)

○ 36+ (37)

Display This Question:

If Please indicate if you have been in contact with any of the following services because of your he... = GP [Yes]

Q264 Please indicate the average length of GP visit
Average length of visit (minutes) (1)

- 15 (1)
- 30 (2)
- 45 (3)
- 60 (4)
- 75 (5)
- 90 (6)
- 105 (7)
- 120 (8)
- 180 (9)
- 240 (10)
- 300 (11)
- 300+ (12)

Display This Question:

If Please indicate if you have been in contact with any of the following services because of your he... = GP Practice Nurse [Yes]

Q265 Please indicate the number of contacts with GP Practice Nurses in the last 3 months
Number of contacts with GP nurses (1)

- ○ 0 (1)
- ○ 1 (2)
- ○ 2 (3)
- ○ 3 (4)
- ○ 4 (5)
- ○ 5 (6)
- ○ 6 (7)
- ○ 7 (8)
- ○ 8 (9)
- ○ 9 (10)
- ○ 10 (11)
- ○ 11 (12)
- ○ 12 (13)
- ○ 13 (14)
- ○ 14 (15)
- ○ 15 (16)
- ○ 16 (17)
- ○ 17 (18)
- ○ 18 (19)
- ○ 19 (20)

○ 20 (21)

○ 21 (22)

○ 22 (23)

○ 23 (24)

○ 24 (25)

○ 25 (26)

○ 26 (27)

○ 27 (28)

○ 28 (29)

○ 29 (30)

○ 30 (31)

○ 31 (32)

○ 32 (33)

○ 33 (34)

○ 34 (35)

○ 35 (36)

○ 36+ (37)

Display This Question:

If Please indicate if you have been in contact with any of the following services because of your he... = GP Practice Nurse [Yes]

Q266 Please indicate the average length of contacts with GP Nurses in the last 3 months
Average length of contact with GP Nurses (minutes) (4)

- 15 (1)
- 30 (2)
- 45 (3)
- 60 (4)
- 75 (5)
- 90 (6)
- 105 (7)
- 120 (8)
- 180 (9)
- 240 (10)
- 300 (11)
- 300+ (12)

End of Block: Impacts on carer health_rev

Start of Block: Psychologist_rev

Display This Question:

If Please indicate if you have been in contact with any of the following services because of your he... = Psychologist [Yes]

Q267 Please indicate the number of contacts with psychologists in the last 3 months
Number of contacts with psychologists (1)

- 0 (1)
- 1 (2)
- 2 (3)
- 3 (4)
- 4 (5)
- 5 (6)
- 6 (7)
- 7 (8)
- 8 (9)
- 9 (10)
- 10 (11)
- 11 (12)
- 12 (13)
- 13 (14)
- 14 (15)
- 15 (16)
- 16 (17)
- 17 (18)
- 18 (19)
- 19 (20)

○ 20 (21)

○ 21 (22)

○ 22 (23)

○ 23 (24)

○ 24 (25)

○ 25 (26)

○ 26 (27)

○ 27 (28)

○ 28 (29)

○ 29 (30)

○ 30 (31)

○ 31 (32)

○ 32 (33)

○ 33 (34)

○ 34 (35)

○ 35 (36)

○ 36+ (37)

Display This Question:

If Please indicate if you have been in contact with any of the following services because of your he... = Psychologist [Yes]

Q268 Please indicate the average length of contact with psychologists
Average length of contact (minutes) (4)

- 15 (1)
- 30 (2)
- 45 (3)
- 60 (4)
- 75 (5)
- 90 (6)
- 105 (7)
- 120 (8)
- 180 (9)
- 240 (10)
- 300 (11)
- 300+ (12)

End of Block: Psychologist_rev

Start of Block: Physiotherapist_rev

Display This Question:

If Please indicate if you have been in contact with any of the following services because of your he... = Phsyiotherapist [Yes]

Q280 Please indicate the number of contacts with physiotherapists in the last 3 months
Number of contacts with physiotherapists (1)

- ○ 0 (1)
- ○ 1 (2)
- ○ 2 (3)
- ○ 3 (4)
- ○ 4 (5)
- ○ 5 (6)
- ○ 6 (7)
- ○ 7 (8)
- ○ 8 (9)
- ○ 9 (10)
- ○ 10 (11)
- ○ 11 (12)
- ○ 12 (13)
- ○ 13 (14)
- ○ 14 (15)
- ○ 15 (16)
- ○ 16 (17)
- ○ 17 (18)
- ○ 18 (19)
- ○ 19 (20)

20 (21)

21 (22)

22 (23)

23 (24)

24 (25)

25 (26)

26 (27)

27 (28)

28 (29)

29 (30)

30 (31)

31 (32)

32 (33)

33 (34)

34 (35)

35 (36)

36+ (37)

Display This Question:

If Please indicate if you have been in contact with any of the following services because of your he... = Phsyiotherapist [Yes]

Q281 Please indicate the average length of contact with physiotherapists.
Average length of contact (minutes) (4)

- 15 (1)
- 30 (2)
- 45 (3)
- 60 (4)
- 75 (5)
- 90 (6)
- 105 (7)
- 120 (8)
- 180 (9)
- 240 (10)
- 300 (11)
- 300+ (12)

End of Block: Physiotherapist_rev

Start of Block: Occupational therapist_rev

Display This Question:

If Please indicate if you have been in contact with any of the following services because of your he... = Occupational Therapist [Yes]

Q282 Please indicate the number of contacts with occupational therapists in the last 3 months
Number of contacts with occupational therapists (1)

- 0 (1)
- 1 (2)
- 2 (3)
- 3 (4)
- 4 (5)
- 5 (6)
- 6 (7)
- 7 (8)
- 8 (9)
- 9 (10)
- 10 (11)
- 11 (12)
- 12 (13)
- 13 (14)
- 14 (15)
- 15 (16)
- 16 (17)
- 17 (18)
- 18 (19)
- 19 (20)

○ 20 (21)

○ 21 (22)

○ 22 (23)

○ 23 (24)

○ 24 (25)

○ 25 (26)

○ 26 (27)

○ 27 (28)

○ 28 (29)

○ 29 (30)

○ 30 (31)

○ 31 (32)

○ 32 (33)

○ 33 (34)

○ 34 (35)

○ 35 (36)

○ 36+ (37)

Display This Question:

If Please indicate if you have been in contact with any of the following services because of your he... = Occupational Therapist [Yes]

Q283 Please indicate the average length of contact with occupational therapists.
Average length of contact (minutes) (4)

- 15 (1)
- 30 (2)
- 45 (3)
- 60 (4)
- 75 (5)
- 90 (6)
- 105 (7)
- 120 (8)
- 180 (9)
- 240 (10)
- 300 (11)
- 300+ (12)

End of Block: Occupational therapist_rev

Start of Block: Community Mental Health Team_rev

Display This Question:

If Please indicate if you have been in contact with any of the following services because of your he... = Community Mental Health Team [Yes]

Q284 Please indicate the number of contacts in the last 3 months with community mental health teams.
Number of contacts with community mental health teams (1)

- 0 (1)
- 1 (2)
- 2 (3)
- 3 (4)
- 4 (5)
- 5 (6)
- 6 (7)
- 7 (8)
- 8 (9)
- 9 (10)
- 10 (11)
- 11 (12)
- 12 (13)
- 13 (14)
- 14 (15)
- 15 (16)
- 16 (17)
- 17 (18)
- 18 (19)
- 19 (20)

- ○ 20 (21)
- ○ 21 (22)
- ○ 22 (23)
- ○ 23 (24)
- ○ 24 (25)
- ○ 25 (26)
- ○ 26 (27)
- ○ 27 (28)
- ○ 28 (29)
- ○ 29 (30)
- ○ 30 (31)
- ○ 31 (32)
- ○ 32 (33)
- ○ 33 (34)
- ○ 34 (35)
- ○ 35 (36)
- ○ 36+ (37)

Display This Question:

If Please indicate if you have been in contact with any of the following services because of your he... = Community Mental Health Team [Yes]

Q285 Please indicate the average length of contact with community mental health teams.
Average length of contact with community mental health teams (minutes) (1)

- 15 (1)
- 30 (2)
- 45 (3)
- 60 (4)
- 75 (5)
- 90 (6)
- 105 (7)
- 120 (8)
- 180 (9)
- 240 (10)
- 300 (11)
- 300+ (12)

End of Block: Community Mental Health Team_rev

Start of Block: Social worker_rev

Display This Question:

If Please indicate if you have been in contact with any of the following services because of your he... = Social Worker [Yes]

Q286 Please indicate the number of contacts with social workers in the last 3 months.
Number of contacts with social workers (1)

- 0 (1)
- 1 (2)
- 2 (3)
- 3 (4)
- 4 (5)
- 5 (6)
- 6 (7)
- 7 (8)
- 8 (9)
- 9 (10)
- 10 (11)
- 11 (12)
- 12 (13)
- 13 (14)
- 14 (15)
- 15 (16)
- 16 (17)
- 17 (18)
- 18 (19)
- 19 (20)

◯ 20 (21)

◯ 21 (22)

◯ 22 (23)

◯ 23 (24)

◯ 24 (25)

◯ 25 (26)

◯ 26 (27)

◯ 27 (28)

◯ 28 (29)

◯ 29 (30)

◯ 30 (31)

◯ 31 (32)

◯ 32 (33)

◯ 33 (34)

◯ 34 (35)

◯ 35 (36)

◯ 36+ (37)

Display This Question:

If Please indicate if you have been in contact with any of the following services because of your he... = Social Worker [Yes]

Q287 Please indicate the average length of contact with social workers.
Average length of contact with social workers (minutes) (4)

○ 15 (1)

○ 30 (2)

○ 45 (3)

○ 60 (4)

○ 75 (5)

○ 90 (6)

○ 105 (7)

○ 120 (8)

○ 180 (9)

○ 240 (10)

○ 300 (11)

○ 300+ (12)

End of Block: Social worker_rev

Start of Block: Cam therapist_rev

Display This Question:

If Please indicate if you have been in contact with any of the following services because of your he... = Complementary / Alternative Medicine (e.g. Acupuncture, Mindfulness, Yoga etc) [Yes]

Q292 Please indicate the number of contacts in the last 3 months with complementary / alternative medicine therapists.
Number of contacts with complementary / alternative medicine therapists (1)

- 0 (1)
- 1 (2)
- 2 (3)
- 3 (4)
- 4 (5)
- 5 (6)
- 6 (7)
- 7 (8)
- 8 (9)
- 9 (10)
- 10 (11)
- 11 (12)
- 12 (13)
- 13 (14)
- 14 (15)
- 15 (16)
- 16 (17)
- 17 (18)
- 18 (19)
- 19 (20)

○ 20 (21)

○ 21 (22)

○ 22 (23)

○ 23 (24)

○ 24 (25)

○ 25 (26)

○ 26 (27)

○ 27 (28)

○ 28 (29)

○ 29 (30)

○ 30 (31)

○ 31 (32)

○ 32 (33)

○ 33 (34)

○ 34 (35)

○ 35 (36)

○ 36+ (37)

Display This Question:

If Please indicate if you have been in contact with any of the following services because of your he... = Complementary / Alternative Medicine (e.g. Acupuncture, Mindfulness, Yoga etc) [Yes]

Q293 Please indicate the average length of contact with complementary / alternative medicine therapists.
Average length of contact with complementary / alternative therapists (minutes) (4)

- 1 (1)
- 1 ~ 15 (2)
- 1 ~ 30 (3)
- 1 ~ 45 (4)
- 1 ~ 60 (5)
- 1 ~ 75 (6)
- 1 ~ 90 (7)
- 1 ~ 105 (8)
- 1 ~ 120 (9)
- 2 (10)
- 2 ~ 15 (11)
- 2 ~ 30 (12)
- 2 ~ 45 (13)
- 2 ~ 60 (14)
- 2 ~ 75 (15)
- 2 ~ 90 (16)
- 2 ~ 105 (17)
- 2 ~ 120 (18)
- 3 (19)
- 3 ~ 15 (20)

- 3 ~ 30 (21)
- 3 ~ 45 (22)
- 3 ~ 60 (23)
- 3 ~ 75 (24)
- 3 ~ 90 (25)
- 3 ~ 105 (26)
- 3 ~ 120 (27)
- 4 (28)
- 4 ~ 15 (29)
- 4 ~ 30 (30)
- 4 ~ 45 (31)
- 4 ~ 60 (32)
- 4 ~ 75 (33)
- 4 ~ 90 (34)
- 4 ~ 105 (35)
- 4 ~ 120 (36)
- 5 (37)
- 5 ~ 15 (38)
- 5 ~ 30 (39)
- 5 ~ 45 (40)
- 5 ~ 60 (41)

- 5 ~ 75 (42)
- 5 ~ 90 (43)
- 5 ~ 105 (44)
- 5 ~ 120 (45)
- 6 (46)
- 6 ~ 15 (47)
- 6 ~ 30 (48)
- 6 ~ 45 (49)
- 6 ~ 60 (50)
- 6 ~ 75 (51)
- 6 ~ 90 (52)
- 6 ~ 105 (53)
- 6 ~ 120 (54)
- 7 (55)
- 7 ~ 15 (56)
- 7 ~ 30 (57)
- 7 ~ 45 (58)
- 7 ~ 60 (59)
- 7 ~ 75 (60)
- 7 ~ 90 (61)
- 7 ~ 105 (62)

- 7 ~ 120 (63)
- 8 (64)
- 8 ~ 15 (65)
- 8 ~ 30 (66)
- 8 ~ 45 (67)
- 8 ~ 60 (68)
- 8 ~ 75 (69)
- 8 ~ 90 (70)
- 8 ~ 105 (71)
- 8 ~ 120 (72)
- 9 (73)
- 9 ~ 15 (74)
- 9 ~ 30 (75)
- 9 ~ 45 (76)
- 9 ~ 60 (77)
- 9 ~ 75 (78)
- 9 ~ 90 (79)
- 9 ~ 105 (80)
- 9 ~ 120 (81)
- 10+ (82)
- 10+ ~ 15 (83)

○ 10+ ~ 30 (84)

○ 10+ ~ 45 (85)

○ 10+ ~ 60 (86)

○ 10+ ~ 75 (87)

○ 10+ ~ 90 (88)

○ 10+ ~ 105 (89)

○ 10+ ~ 120 (90)

End of Block: Cam therapist_rev

Start of Block: Other therapist_rev

Display This Question:

If Please indicate if you have been in contact with any of the following services because of your he... = Other therapist [Yes]

Q294 Please indicate the number of contacts in the last 3 months with other therapists.
Number of contacts with other therapists (1)

- 0 (1)
- 1 (2)
- 2 (3)
- 3 (4)
- 4 (5)
- 5 (6)
- 6 (7)
- 7 (8)
- 8 (9)
- 9 (10)
- 10 (11)
- 11 (12)
- 12 (13)
- 13 (14)
- 14 (15)
- 15 (16)
- 16 (17)
- 17 (18)
- 18 (19)
- 19 (20)

- ○ 20 (21)
- ○ 21 (22)
- ○ 22 (23)
- ○ 23 (24)
- ○ 24 (25)
- ○ 25 (26)
- ○ 26 (27)
- ○ 27 (28)
- ○ 28 (29)
- ○ 29 (30)
- ○ 30 (31)
- ○ 31 (32)
- ○ 32 (33)
- ○ 33 (34)
- ○ 34 (35)
- ○ 35 (36)
- ○ 36+ (37)

Display This Question:

If Please indicate if you have been in contact with any of the following services because of your he... = Other therapist [Yes]

Q295 Please indicate the average length of contact with other therapists.
Average length of contact with other therapists (minutes) (4)

- ○ 15 (1)
- ○ 30 (2)
- ○ 45 (3)
- ○ 60 (4)
- ○ 75 (5)
- ○ 90 (6)
- ○ 105 (7)
- ○ 120 (8)
- ○ 180 (9)
- ○ 240 (10)
- ○ 300 (11)
- ○ 300+ (12)

End of Block: Other therapist_rev

Start of Block: hospital visits_rev

Display This Question:

If Please indicate if you have been in contact with any of the following services because of your he... = Visits to hospital not involving an overnight stay [Yes]

Q296 Please indicate the number of A & E, planned outpatient and other hospital visits that did not involve an overnight stay in the last 3 months

	1 (undefined)	2 (21)	3 (22)	4 (23)	5 (24)	6 (25)	7 (26)	8 (27)	9 (28)	10 (29)	11 (30)	12 (31)	13 (32)	14 (33)	(
Number of A & E visits (1)															
Number of of planned outpatient visits (6)															
Number of other visits (e.g. for screenings / blood tests) (7)															

End of Block: hospital visits_rev

Start of Block: Hospital inpatient_rev

Display This Question:

If Please indicate if you have been in contact with any of the following services because of your he... = Visits to hospital involving an overnight stay [Yes]

Q297 Please indicate how many times you were admitted to hospital in the last 3 months.
Total number of hospital admissions (1)

- ○ 0 (1)
- ○ 1 (2)
- ○ 2 (3)
- ○ 3 (4)
- ○ 4 (5)
- ○ 5 (6)
- ○ 6 (7)
- ○ 7 (8)
- ○ 8 (9)
- ○ 9 (10)
- ○ 10 (11)
- ○ 11 (12)
- ○ 12 (13)
- ○ 13 (14)
- ○ 14 (15)
- ○ 15 (16)
- ○ 16 (17)
- ○ 17 (18)
- ○ 18 (19)
- ○ 19 (20)

○ 20 (21)

○ 21 (22)

○ 22 (23)

○ 23 (24)

○ 24 (25)

○ 25 (26)

○ 26 (27)

○ 27 (28)

○ 28 (29)

○ 29 (30)

○ 30 (31)

○ 31 (32)

○ 32 (33)

○ 33 (34)

○ 34 (35)

○ 35 (36)

○ 36+ (37)

Display This Question:

If Please indicate if you have been in contact with any of the following services because of your he... = Visits to hospital involving an overnight stay [Yes]

Q298 Please indicate the total number of nights spent in hospital in the last 3 months
Total number of nights spent in hospital (4)

- 1 (1)
- 2 (2)
- 3 (3)
- 4 (4)
- 5 (5)
- 6 (6)
- 7 (7)
- 8 (8)
- 9 (9)
- 10+ (10)
- 20+ (11)
- 30+ (12)
- 60+ (13)
- 90+ (14)

End of Block: Hospital inpatient_rev

Start of Block: Paid carers_rev

Q299 Please indicate which of these statements apply to you

	Yes (1)	No (2)
I have requested or had a carer's assessment (1)	○	○
I receive the Carer's Allowance (2)	○	○
I receive support from paid carers (4)	○	○
The person i care for attends support activities (6)	○	○
I take part in a support group (5)	○	○
I receive other support with caring (7)	○	○

Display This Question:

If Please indicate which of these statements apply to you = I receive support from paid carers [Yes]

Q300 Please indicate the weekly number of contacts with paid carers
Number of weekly contacts with paid carers (1)

- ○ 0 (1)
- ○ 1 (2)
- ○ 2 (3)
- ○ 3 (4)
- ○ 4 (5)
- ○ 5 (6)
- ○ 6 (7)
- ○ 7 (8)
- ○ 8 (9)
- ○ 9 (10)
- ○ 10 (11)
- ○ 11 (12)
- ○ 12 (13)
- ○ 13 (14)
- ○ 14 (15)
- ○ 15 (16)
- ○ 16 (17)
- ○ 17 (18)
- ○ 18 (19)
- ○ 19 (20)

- 20 (21)
- 21 (22)
- 22 (23)
- 23 (24)
- 24 (25)
- 25 (26)
- 26 (27)
- 27 (28)
- 28 (29)
- 29 (30)
- 30 (31)
- 31 (32)
- 32 (33)
- 33 (34)
- 34 (35)
- 35 (36)
- 36+ (37)

Display This Question:

If Please indicate which of these statements apply to you = I receive support from paid carers [Yes]

Q301 Please indicate the average length of contacts with paid carers
Average length of contact (minutes) (4)

- ◯ 15 (1)
- ◯ 30 (2)
- ◯ 45 (3)
- ◯ 60 (4)
- ◯ 75 (5)
- ◯ 90 (6)
- ◯ 105 (7)
- ◯ 120 (8)
- ◯ 180 (9)
- ◯ 240 (10)
- ◯ 300 (11)
- ◯ 300+ (12)

End of Block: Paid carers_rev

Start of Block: supported activities outside home_rev

Display This Question:

If Please indicate which of these statements apply to you = The person i care for attends support activities [Yes]

Q302 Please indicate how many days in a typical week the person you support attends external supported activities
Number of days per week attending external activities (1)

- ○ 1 (1)
- ○ 2 (2)
- ○ 3 (3)
- ○ 4 (4)
- ○ 5 (5)
- ○ 6 (6)
- ○ 7 (7)

Display This Question:

If Please indicate which of these statements apply to you = I receive other support with caring [Yes]

Q303 Please indicate the average length of external support activities for the person you care for
Average length of contact for external activities (minutes) (4)

- ○ 15 (1)
- ○ 30 (2)
- ○ 45 (3)
- ○ 60 (4)
- ○ 75 (5)
- ○ 90 (6)
- ○ 105 (7)
- ○ 120 (8)
- ○ 180 (9)
- ○ 240 (10)
- ○ 300 (11)
- ○ 300+ (12)

End of Block: supported activities outside home_rev

Start of Block: other carer support

Display This Question:

If Please indicate which of these statements apply to you = I take part in a support group [Yes]

Q90 Please indicate how many hours you spend in peer support groups in a typical week
Number of hours of peer support per week (1)

- 1 (1)
- 1 ~ 15 minutes (2)
- 1 ~ 30 minutes (3)
- 1 ~ 45 minutes (4)
- 1 ~ 60 minutes (5)
- 1 ~ 75 minutes (6)
- 1 ~ 90 minutes (7)
- 1 ~ 105 minutes (8)
- 1 ~ 2 hours (9)
- 1 ~ 3 hours (10)
- 1 ~ 4 hours (11)
- 1 ~ 5 hours + (12)
- 2 (13)
- 2 ~ 15 minutes (14)
- 2 ~ 30 minutes (15)
- 2 ~ 45 minutes (16)
- 2 ~ 60 minutes (17)
- 2 ~ 75 minutes (18)
- 2 ~ 90 minutes (19)
- 2 ~ 105 minutes (20)

- 2 ~ 2 hours (21)
- 2 ~ 3 hours (22)
- 2 ~ 4 hours (23)
- 2 ~ 5 hours + (24)
- 3 (25)
- 3 ~ 15 minutes (26)
- 3 ~ 30 minutes (27)
- 3 ~ 45 minutes (28)
- 3 ~ 60 minutes (29)
- 3 ~ 75 minutes (30)
- 3 ~ 90 minutes (31)
- 3 ~ 105 minutes (32)
- 3 ~ 2 hours (33)
- 3 ~ 3 hours (34)
- 3 ~ 4 hours (35)
- 3 ~ 5 hours + (36)
- 4 (37)
- 4 ~ 15 minutes (38)
- 4 ~ 30 minutes (39)
- 4 ~ 45 minutes (40)
- 4 ~ 60 minutes (41)

- 4 ~ 75 minutes (42)
- 4 ~ 90 minutes (43)
- 4 ~ 105 minutes (44)
- 4 ~ 2 hours (45)
- 4 ~ 3 hours (46)
- 4 ~ 4 hours (47)
- 4 ~ 5 hours + (48)
- 5 (49)
- 5 ~ 15 minutes (50)
- 5 ~ 30 minutes (51)
- 5 ~ 45 minutes (52)
- 5 ~ 60 minutes (53)
- 5 ~ 75 minutes (54)
- 5 ~ 90 minutes (55)
- 5 ~ 105 minutes (56)
- 5 ~ 2 hours (57)
- 5 ~ 3 hours (58)
- 5 ~ 4 hours (59)
- 5 ~ 5 hours + (60)
- 6 (61)
- 6 ~ 15 minutes (62)

- 6 ~ 30 minutes (63)
- 6 ~ 45 minutes (64)
- 6 ~ 60 minutes (65)
- 6 ~ 75 minutes (66)
- 6 ~ 90 minutes (67)
- 6 ~ 105 minutes (68)
- 6 ~ 2 hours (69)
- 6 ~ 3 hours (70)
- 6 ~ 4 hours (71)
- 6 ~ 5 hours + (72)
- 7 (73)
- 7 ~ 15 minutes (74)
- 7 ~ 30 minutes (75)
- 7 ~ 45 minutes (76)
- 7 ~ 60 minutes (77)
- 7 ~ 75 minutes (78)
- 7 ~ 90 minutes (79)
- 7 ~ 105 minutes (80)
- 7 ~ 2 hours (81)
- 7 ~ 3 hours (82)
- 7 ~ 4 hours (83)

- 7 ~ 5 hours + (84)
- 8 (85)
- 8 ~ 15 minutes (86)
- 8 ~ 30 minutes (87)
- 8 ~ 45 minutes (88)
- 8 ~ 60 minutes (89)
- 8 ~ 75 minutes (90)
- 8 ~ 90 minutes (91)
- 8 ~ 105 minutes (92)
- 8 ~ 2 hours (93)
- 8 ~ 3 hours (94)
- 8 ~ 4 hours (95)
- 8 ~ 5 hours + (96)
- 9 (97)
- 9 ~ 15 minutes (98)
- 9 ~ 30 minutes (99)
- 9 ~ 45 minutes (100)
- 9 ~ 60 minutes (101)
- 9 ~ 75 minutes (102)
- 9 ~ 90 minutes (103)
- 9 ~ 105 minutes (104)

○ 9 ~ 2 hours (105)

○ 9 ~ 3 hours (106)

○ 9 ~ 4 hours (107)

○ 9 ~ 5 hours + (108)

○ 10+ (109)

○ 10+ ~ 15 minutes (110)

○ 10+ ~ 30 minutes (111)

○ 10+ ~ 45 minutes (112)

○ 10+ ~ 60 minutes (113)

○ 10+ ~ 75 minutes (114)

○ 10+ ~ 90 minutes (115)

○ 10+ ~ 105 minutes (116)

○ 10+ ~ 2 hours (117)

○ 10+ ~ 3 hours (118)

○ 10+ ~ 4 hours (119)

○ 10+ ~ 5 hours + (120)

End of Block: other carer support

Start of Block: miscellaneous carer support

Start of Block: Section B: Understanding time spent caring

Q152 Thinking about a typical week please indicate how many hours a week you spend on the following activities:

	0 hours (1)	1-5 hours (26)	6 -10 hours (27)	11 - 15 hours (28)	16 - 20 hours (29)	21 - 25 hours (30)	26 - 30 hours (31)	31 - 40 hours (32)	41 - 50 hours (33)	51 - 60 hours (34)	60 - 80 hours (35)	80 - 100 hour (36
Supervising and monitoring activities (1)												
Emotional support and encouragement (encouraging, motivating, providing intensive emotional support and companionship) (4)												
Responding to challenging behaviour (managing crises, managing inappropriate behaviours) (5)												
Other emotional support or psychosocial care (6)												
Household tasks (grocery shopping, preparing meals, housework, property maintenance) (7)												
Health care coordination. (supervising or												

prompting medication, arranging supervision / outside services, assisting and liaising with health professionals, assisting with other aspects of treatment plans) (8)
Managing finances /paying bills, other paper work, reading and writing or communication (9)
Travel-related activities (getting to appointments, workplace) (10)
Other practical tasks (11)
Helping with activities of daily living. (can include personal hygiene, grooming, bathing and showering, dressing, eating and mobility (12)

Q24 Please identify the three activities where you would like to receive help, with 1 being the most important, 2 being the second most important and 3 being the third most important area for help.

_______ Supervising and monitoring (1)
_______ Emotional support and encouragement (2)
_______ Responding to behaviour (3)
_______ Other emotional support or psychosocial care (4)
_______ Household tasks (shopping, preparing meals etc) (5)
_______ Health care coordination (6)
_______ Managing finances/paying bills, other paper work, reading and writing, communication (7)
_______ Transport (8)
_______ Other practical tasks (9)
_______ Activities of daily living (help with washing, dressing, eating etc) (10)

Display This Question:

If Does the person you care for live with you? = No

Q28 In a typical week how much time do you spend travelling to and from the person you support's place of residence?
Number of hours per week (4)

- 1 (1)
- 2 (2)
- 3 (3)
- 4 (4)
- 5 (5)
- 6 (6)
- 7 (7)
- 8 (8)
- 9 (9)
- 10 (10)
- 11 (11)
- 12 (12)
- 13 (13)
- 14 (14)
- 15 (15)
- 16 (16)
- 17 (17)
- 18 (18)
- 19 (19)
- 20 (20)

○ 21 (21)

○ 22 (22)

○ 23 (23)

○ 24 (24)

○ 25 (25)

○ 26 (26)

○ 27 (27)

○ 28 (28)

○ 29 (29)

○ 30 (30)

○ 31 (31)

○ 32 (32)

○ 33 (33)

○ 34 (34)

○ 35 (35)

○ 36+ (36)

Display This Question:

If Does the person you care for live with you? = No

Q30 In a typical week how many times would you visit the person you support?
Number of visits per week (4)

○ 1 (1)

○ 2 (2)

○ 3 (3)

○ 4 (4)

○ 5 (5)

○ 6 (6)

○ 7 (7)

○ 8 (8)

○ 9 (9)

○ 10+ (10)

Display This Question:

If Does the person you care for live with you? = No

Q31 Please estimate your travel costs for a return journey to visit them?
Costs for a return journey (£s) (4)

- ○ Less than 5 pounds (1)
- ○ 5 - 10 pounds (2)
- ○ 11 - 15 pounds (3)
- ○ 16 - 20 pounds (4)
- ○ 21 - 25 pounds (5)
- ○ 26 - 30 pounds (6)
- ○ 31 - 35 pounds (7)
- ○ 36 - 40 pounds (8)
- ○ 41 - 45 pounds (9)
- ○ 46 - 50 pounds (10)
- ○ More than 50 pounds (11)

End of Block: Section B: Understanding time spent caring

Start of Block: finances and family life

Q126 Thinking about your current circumstances how concerned are you about:

	Not at all (1)	A little (2)	Moderately (3)	Quite a bit (4)	A lot (5)
Your finances (1)	○	○	○	○	○
The finances of the person you care for (2)	○	○	○	○	○
Extra regular costs of caring (4)	○	○	○	○	○
Unexpected crisis costs of caring (5)	○	○	○	○	○
Your career, including promotion opportunities (6)	○	○	○	○	○
Your retirement income (7)	○	○	○	○	○

Q228 Thinking about your retirement do you think you have had or will have to postpone your retirement because of your caring responsibilities?

○ Yes (1)

○ No (2)

Q229 Have your caring responsibilities had an impact on the quality of your relationship with your family?

- Strengthened a lot (1)
- Strengthened a little (2)
- No impact (3)
- Weakened a little (4)
- Weakened a lot (5)

End of Block: finances and family life

Start of Block: About the carer

Q2 We would like to ask a few more questions about your situation. Are you caring for more than one person with mental health problems?

- Yes (1)
- No (2)

Q239 How would you describe your ethnic origin?

- English/Welsh/Scottish/Northern Irish/British (1)
- Irish (2)
- Gypsy or Irish Traveller (3)
- Any other white background (4)
- White and Black Carribbean (6)
- White and Black African (7)
- White and Asian (8)
- Any other mixed background (9)
- Indian (10)
- Pakistani (11)
- Bangladeshi (12)
- Chinese (13)
- Caribbean (14)
- African (15)
- Any other black background (16)
- Arab (5)
- Any other ethnic background (17)
- Prefer not to say (18)

Q61 We would like to know a little more about your own health and how this affects your ability to be a carer. Have you been diagnosed with any condition that impacts on your ability to care? (Select ALL that apply)

- ☐ No health condition (1)
- ☐ Arthritis (2)
- ☐ Back Pain (3)
- ☐ Cancer (4)
- ☐ Cardiovascular Disease (5)
- ☐ Diabetes (6)
- ☐ Epilepsy (7)
- ☐ Movement Disorders (e.g. Parkinson's Disease, Huntingdon's Disease, Cerebal Palsy) (8)
- ☐ Permanent Disabilities including Hearing Loss and Visual Impairment (9)
- ☐ Respiratory Disease (e.g. Asthma, Chronic Obstructive Pulmonary Disease – COPD) (10)
- ☐ Depression and / or Anxiety Disorders (13)
- ☐ Other (11)
- ☐ Prefer not to say (12)

End of Block: About the carer

Start of Block: Carer education and other info

Q102 What is the highest level of qualification that you have received from school, college or since leaving education? Please include any work-based training.

- ○ Degree level qualification (or equivalent) (1)
- ○ Higher education qualification below degree level (2)
- ○ A-Levels or Highers or Advanced Diploma or Progression Diploma (3)
- ○ ONC / National Level BTEC (4)
- ○ O-Level /GCSE Grade A-C; CSE Grade 1; Standard Grade 1 -3; Higher Diploma (5)
- ○ GCSE grade D-G or CSE grade 2-5 or Standard Grade 4-6 or Foundation Diploma (6)
- ○ Other qualifications (including foreign qualifications below degree level) (7)
- ○ No formal qualifications (8)

Q103 We would like to know more about where you live.

- ○ Owner occupied house or flat (1)
- ○ Privately rented house or flat (2)
- ○ Public sector or housing association/cooperative rented house or flat (3)
- ○ Other (4)

Q104 Urban / Rural - Do you live in an area with

- ○ More than 125,000 people (1)
- ○ Between 10,001 and 125,000 people (2)
- ○ Between 3000 and 10,000 people (3)
- ○ Less than 3000 people (4)

Q135 Which professional category best fits you?

- ○ Manager (1)
- ○ Professional (e.g. Scientists, Medics, Teachers, IT, Legal, Business, Adminstration). (2)
- ○ Technician or Associated Professional (3)
- ○ Clerical Support Worker (4)
- ○ Services and Sales Worker (e.g. Cook, Waiter, Hairdresser, Care Worker) (5)
- ○ Skilled agricultural, forestry and fishery workers (6)
- ○ Craft and related trades workers (7)
- ○ Plant and machine operators, and assemblers (8)
- ○ Elementary Occupations (e.g. Cleaners, General Labourers, Refuse Workers) (9)
- ○ Armed forces (10)

End of Block: Carer education and other info

Start of Block: Carer income question

Q112 What is your gross income from all sources before tax and national insurance?

- Up to £100 per week (1)
- £101 to £200 per week (2)
- £201 to £300 per week (3)
- £301 to £400 per week (4)
- £401 to £500 per week (5)
- £501 to £600 per week (6)
- £601 to £700 per week (7)
- £701 to £800 per week (8)
- £801 to £900 per week (9)
- £901 to £1000 per week (10)
- More than £1000 per week (11)
- Prefer not to say (12)
- Dont know (13)

End of Block: Carer income question

Start of Block: Use of hospital by the person being cared for_rev

Q269 We would like to know some more about the health of the person you care for and how this impacts on caring. Has the person you care for been diagnosed with any other **physical health** conditions that requires significant care? (Select ALL that apply)

- ☐ No, no other health condition (1)
- ☐ Cancer (2)
- ☐ Cardiovascular Disease (3)
- ☐ Diabetes (4)
- ☐ Dementia (5)
- ☐ Epilepsy (6)
- ☐ Movement Disorders (e.g. Parkinson's Disease, Huntington's Disease, Cerebral Palsy, etc.) (7)
- ☐ Permanent disabilities including Hearing Loss and Visual Impairment (8)
- ☐ Respiratory Disease (e.g. Asthma, Chronic Obstructive Pulmonary Disease - COPD) (9)
- ☐ Other (10)
- ☐ Dont know (11)

Q271 Has the person you care for had an overnight stay in hospital in the last 3 months because of their mental health?

- ○ Yes (1)
- ○ No (2)

Display This Question:

If Has the person you care for had an overnight stay in hospital in the last 3 months because of the... = Yes

Q272 Please specify how many times they have been admitted to hospital in the last 3 months because of their mental health and the total number of nights spent in hospital
Total number of hospital admissions (4)

- ○ 0 (1)
- ○ 1 (2)
- ○ 2 (3)
- ○ 3 (4)
- ○ 4 (5)
- ○ 5 (6)
- ○ 6 (7)
- ○ 7 (8)
- ○ 8 (9)
- ○ 9 (10)
- ○ 10 (11)
- ○ 11 (12)
- ○ 12 (13)
- ○ 13 (14)
- ○ 14 (15)
- ○ 15 (16)
- ○ 16 (17)
- ○ 17 (18)
- ○ 18 (19)
- ○ 19 (20)

- 20 (21)
- 21 (22)
- 22 (23)
- 23 (24)
- 24 (25)
- 25 (26)
- 26 (27)
- 27 (28)
- 28 (29)
- 29 (30)
- 30 (31)
- 31 (32)
- 32 (33)
- 33 (34)
- 34 (35)
- 35 (36)
- 36+ (37)

Display This Question:

If Has the person you care for had an overnight stay in hospital in the last 3 months because of the... = Yes

Q273 Please specify the total number of nights the person you care for spent in hospital in the last 3 months because of their mental health
Total number of nights in hospital (5)

- ○ 1 (1)
- ○ 2 (2)
- ○ 3 (3)
- ○ 4 (4)
- ○ 5 (5)
- ○ 6 (6)
- ○ 7 (7)
- ○ 8 (8)
- ○ 9 (9)
- ○ 10+ (10)
- ○ 20+ (11)
- ○ 30+ (12)
- ○ 60+ (13)
- ○ 90+ (14)

Q274 Since you have been caring what is the most frequent cause of unplanned visits to hospital related to mental health of the person you care for? Select **ONE** option only.

- ◯ Not applicable - there have been no unplanned visits to hospital (1)
- ◯ Self-harm and / or suicidal behaviour (2)
- ◯ Alcohol, drugs and other substance abuse (4)
- ◯ Depression (5)
- ◯ Psychosis (6)
- ◯ Agitation (7)
- ◯ Other rapid change in behaviour (3)
- ◯ Other reason (8)

Q276 Does the person you care for experience periods of agitation?

- ◯ Yes (1)
- ◯ No (2)

Display This Question:

If Does the person you care for experience periods of agitation? = Yes

Q277 Please indicate how often they experience episodes of agitation

- At least once a week (1)
- At least once a month (2)
- At least once every 3 months (3)
- At least once a year (4)
- Less often (5)

Display This Question:

If Does the person you care for experience periods of agitation? = Yes

Q278 If the person you care for requires urgent care due to agitation, on average how many extra hours of care do you have to provide for a period of agitation?
Number of hours (4)

- 0 (1)
- 1 (2)
- 2 (3)
- 3 (4)
- 4 (5)
- 5 (6)
- 6 (7)
- 7 (8)
- 8 (9)
- 9 (10)
- 10 (11)
- 11 (12)
- 12 (13)
- 13 (14)
- 14 (15)
- 15 (16)
- 16 (17)
- 17 (18)
- 18 (19)
- 19 (20)

○ 20 (21)

○ 21 (22)

○ 22 (23)

○ 23 (24)

○ 24 (25)

○ 25 (26)

○ 26 (27)

○ 27 (28)

○ 28 (29)

○ 29 (30)

○ 30 (31)

○ 31 (32)

○ 32 (33)

○ 33 (34)

○ 34 (35)

○ 35 (36)

○ 36+ (37)

Q279 On a scale between 0 and 100 when 0 means the worst mental health state you could imagine and 100 means the best mental health state, how would you rate the mental health of the person you care for at the moment?

	Worst Mental Health State	Best mental health state

	0 10 20 30 40 50 60 70 80 90 100
1 ()	

End of Block: Use of hospital by the person being cared for_rev

Start of Block: Final question block

Q259 On a scale between 0 and 100 when 0 means the worst physical health state you could imagine and 100 means the best physical health state, how would you rate the physical health of the person you care for at the moment?

	Worst Physical Health State / Best physical health state<br>0 10 20 30 40 50 60 70 80 90 100
1 ()	

End of Block: Final question block
